# Pan-cancer profiles of mesothelin (MSLN) unveil diagnostic potential and therapeutic targetability of pancreatic adenocarcinoma

**DOI:** 10.1371/journal.pone.0354334

**Published:** 2026-08-20

**Authors:** Md Ramjan Sheikh, Baishakhi Mistry, Ridika Haque Shafin, Amreeta Apu, Tasnia Hossain Sara, Jarin Tasnim, Dipankar Dutta, Aishwarya Nandy, Anisur Rahman

**Affiliations:** 1 Department of Biochemistry and Molecular Biology, Hajee Mohammad Danesh Science and Technology University, Dinajpur, Bangladesh; 2 Department of Biotechnology and Genetic Engineering, Khulna University, Khulna, Bangladesh; 3 Department of Botany, University of Dhaka, Dhaka, Bangladesh; 4 Department of Genetic Engineering and Biotechnology, Jagannath University, Dhaka, Bangladesh; 5 Department of Biochemistry and Molecular Biology, Habibulla Bahar College, Dhaka, Bangladesh; 6 Department of Applied Chemistry and Chemical Engineering, University of Rajshahi, Rajshahi, Bangladesh; 7 Department of Microbiology, University of Dhaka, Dhaka, Bangladesh; 8 Department of Microbiology, Jashore University of Science and Technology, Jashore, Bangladesh; 9 Department of Pharmacy, BGC Trust University, Chattogram, Bangladesh; Kwara State University, NIGERIA

## Abstract

**Background:**

Mesothelin (MSLN) is a cell surface glycoprotein that is often over-expressed in various malignancies of humans and has been suggested as a potential therapeutic and diagnostic target. However, its expression profile, clinical implications and drug-targeting potential are not fully understood and needs to be investigated across various cancer types.

**Objectives:**

The goal of this study was to systematically characterize the MSLN expression pattern, its prognostic value and functionality in different types of cancers, particularly in pancreatic adenocarcinoma (PAAD), as well as to identify viable therapeutic target opportunities.

**Methods:**

Transcriptomic and clinical datasets from TCGA and GEO were used to evaluate the expression of the MSLN gene, diagnostic value, and its prognostic significance. To study the genomic alterations and functional pathways of MSLN, mutation, GO, and KEGG analyses were used. Drug sensitivity correlations were performed with GDSC and CTRP datasets. Structure-based drug discovery involved molecular docking (AutoDock Vina and Maestro Glide), 100 ns molecular dynamics simulations, and MMPBSA binding free energy analysis, followed by drug-likeness and toxicity assessment using SwissADME and ProTox-III.

**Results:**

MSLN was found to be over expressed in several cancers, including PAAD, ovarian and mesothelioma, and this was confirmed in the independent GEO datasets. The analysis of ROC curves showed high accuracy for PAAD and stomach adenocarcinoma and moderate to low accuracy for the other cancers. Both survival and Cox regression analyses showed that MSLN was a cancer-type-specific prognostic factor. Analysis of mutations showed low frequencies of genetic changes, suggesting that the mechanism of dysregulation may be transcriptional or epigenetic. Enrichment analysis highlighted immune-related and tumor-microenvironment signaling pathways associated with MSLN. Drug sensitivity analysis suggested association of MSLN expression with response to targeted and selected chemotherapeutic drugs. Molecular docking revealed Withaferin A as the most potent protein binding compound with MSLN with stable protein-ligand interaction as evidenced by molecular dynamics simulations and binding energy analysis.

**Conclusion:**

MSLN is a diagnosis and prognosis biomarker that is context dependent and has application in selected cancers, especially pancreatic adenocarcinoma. While Withaferin A shows promising in silico binding and stability against MSLN, predicted toxicity and pharmacokinetic limitations suggest the need for further optimization and experimental validation before therapeutic application.

## 1. Introduction

Cancer is one of the leading causes of morbidity and mortality worldwide, accounting for millions of new cases and deaths each year [1]. Although significant progress has been made in cancer diagnostics and targeted therapeutic strategies, the prognosis of some neoplasms, in particular pancreatic adenocarcinoma (PAAD), remain extremely limited, due to their advanced diagnostic time, their rapid metastatic progression, their therapeutic resistance and limited therapeutic options [2]. Despite significant progress, the five-year survival rate for pancreatic cancer remains one of the lowest for any solid tumor, indicating the critical need for biomarkers to help diagnose the disease as well as new targets to help treat it [3].

Mesothelin (MSLN) is a GPI anchored cell surface glycoprotein expressed at low levels in cells of mesothelial origin which is significantly expressed in several human cancers including pancreatic adenocarcinoma, ovarian carcinoma, mesothelioma, lung adenocarcinoma, gastric cancer and triple negative breast cancer [4,5]. MSLN has been shown to be involved in cell adhesion, extracellular communication and oncogenesis signaling pathways, and it appears that MSLN plays a role in tumor progression, tumor invasion, tumor metastasis, and chemoresistance [6]. The most well-characterized association is the binding of MSLN to MUC16 (CA125), a molecule associated with tumor cell adhesion and metastatic spread, especially in pancreatic and ovarian cancer [7]. Due to its tumor-selective overexpression and limited distribution in normal tissues, MSLN has emerged as a promising therapeutic target in oncology. Several MSLN-targeted therapeutic strategies, including monoclonal antibodies, antibody-drug conjugates, immunotoxins, cancer vaccines, and chimeric antigen receptor T-cell (CAR-T) therapies, are currently under investigation [8,9]. But the biological viability of targeting MSLN for the modulation of small molecules is not fully investigated. Small molecules may offer complementary benefits relative to antibody therapeutics, such as tissue penetration, oral bioavailability, reduced manufacturing costs, and engagement of shallow or allosteric interfaces on protein-protein interactions [10].

While individual cancer types have been studied regarding MSLN expression, pan-cancer large scale transcriptomic profiling, diagnostic analysis, prognosis analysis, mutational studies, therapeutic drug sensitivity studies and structure-based therapeutic evaluation of MSLN has been limited, especially in pancreatic adenocarcinoma. In addition, numerous investigations have been conducted regarding the immunotherapeutic use of MSLN with limited number of studies dedicated to the structural druggability and structure-based screening of small molecules against MSLN via computational techniques. Therefore, the present study aimed to perform an integrated pan-cancer investigation of MSLN using publicly available transcriptomic and clinical datasets from The Cancer Genome Atlas (TCGA) and Gene Expression Omnibus (GEO). Differential expression patterns, diagnostic performance, stage-wise associations, survival significance, genomic alterations, and pharmacogenomic correlations were evaluated for MSLN in various types of malignancies. Furthermore, structure-based molecular docking, molecular dynamics simulation, MMPBSA binding energy analysis and ADMET profiling were carried out to generate potential small molecule modulators of MSLN. This multi-layered computational framework aims to understand the biological and therapeutic significance of MSLN with a particular focus on its role in pancreatic adenocarcinoma.

## 2. Materials and methods

### 2.1. Data collection and preprocessing

Transcriptomic expression profiles and corresponding clinical datasets were retrieved from The Cancer Genome Atlas (TCGA) database (https://www.cancer.gov/ccg/research/genome-sequencing/tcga) to investigate the expression pattern and clinical significance of the Mesothelin (MSLN) gene across multiple malignancies [11,12]. Pancreatic adenocarcinoma (PAAD), cholangiocarcinoma (CHOL), stomach adenocarcinoma (STAD), lung adenocarcinoma (LUAD), ovarian serous cystadenocarcinoma (OV), mesothelioma (MESO), and breast invasive carcinoma (BRCA) were analyzed. Selection of the cancer types was based on epithelial or mesothelial relevance, observed MSLN overexpression, availability of matched transcriptomic and clinical data and a known contribution of MSLN to tumor development and therapeutic approaches in solid malignancies such as pancreatic, ovarian, mesothelial and triple-negative breast cancer. To confirm the reproducibility and strength of results, independent external datasets from the Gene Expression Omnibus (GEO) database (https://www.ncbi.nlm.nih.gov/geo/) were obtained [13,14]. The GSE62452 and GSE15471 pancreatic adenocarcinoma cohorts were used as primary and secondary validation datasets, respectively [15,16]. TCGA datasets were used for discovery, and GEO datasets were used as independent external replicates to analysis for cross-dataset replication of MSLN expression patterns. The TCGA-PAAD cohort comprised of 179 samples of tumor tissue and 4 samples of adjacent normal tissue. Likewise, the TCGA-CHOL, TCGA-STAD, TCGA-LUAD, TCGA-OV, TCGA-MESO, and TCGA-BRCA datasets comprised of 36, 415, 524, 379, 87, and 1097 tumor samples, respectively, along with 9, 35, 59, 0, 0, and 113 normal tissue samples, where available. External validation samples comprised 69 pancreatic tumor samples and 61 normal pancreatic tissue samples in GSE62452, whereas GSE15471 contained 39 tumor samples and 39 normal samples. Downstream prognostication was conducted in samples that had no clinical or survival data associated with them. RNA-sequencing and microarray expression data processing were carried out in R studio (v4.4.1) [17]. Standard pre-processing procedures were used to normalize GEO microarray data as log2 counts per million (log2 CPM) to reduce technical variability and allow for comparisons across samples. Data visualization and statistical analyses were performed using the ggplot2, survival, survminer, and pROC packages in R studio (v4.4.1). All datasets were plotted using consistent ranges and color scales to ensure uniformity and reproducibility of the visualization. In this study, no extra ethical approval was sought since all the datasets analyzed were found and obtained through open access public databases. The R scripts for pan-cancer and GEO-based validation analyses are provided in S1 and S2 Files.

### 2.2. Differential expression and pathological stage-wise analysis

Transcriptomic differential expression analysis was done in R studio (v4.4.1) with two-sided Wilcoxon rank-sum test, a non-parametric approach suitable for transcriptomic data that does not necessarily meet normal distribution assumptions [18]. Tumor and adjacent normal tissues were included in the differential expression analysis when normal tissue data were available. Boxplots and violin plots were used to visualize expression distributions to represent median expression and intergroup variations between cancer types. Furthermore, comparative analyses across the different cancers were performed to determine the consistency of MSLN dysregulation across malignancies. To validate MSLN expression patterns found in the discovery cohort of TCGA, external validation analyses were conducted using GSE62452 and GSE15471. Consistent expression trends across TCGA and GEO cohorts were considered supportive of the robustness and reproducibility of MSLN dysregulation. To investigate the association between MSLN expression and tumor progression, stage-stratified analyses were conducted using pathological stage information based on the American Joint Committee on Cancer (AJCC) staging system. The tumor samples were classified based on clinical stage and differences between the clinical stages were compared by the Kruskal-Walli’s test. A stage-wise boxplot was generated to show the trend of expressions related to progression of disease.

### 2.3. Diagnostic performances and survival analysis

The diagnostic performance of MSLN expression was evaluated using Receiver Operating Characteristic (ROC) curve analysis in R studio (v4.4.1) using the pROC package. To assess the discriminatory capacity of MSLN expression between selected cancer types and validation sets, ROC analyses were carried out using expression values of MSLN for both tumor and normal tissue. The quantitative measure of diagnostic performance was expressed as the Area Under the Curve (AUC) and 95% Confidence Interval (CI). Comparative ROC summary plots and bubble visualizations were generated to compare the diagnostic efficiency across cancer types. The prognostic significance of MSLN expression was evaluated using Kaplan-Meier survival analysis [19]. Overall survival (OS) was used as the primary survival endpoint. Survival analyses were performed using tumor samples only, as survival information for normal tissues was unavailable. Patients with expression value above the median value were grouped as the high-expression group and those with expression value below the median were grouped as the low-expression group. For overall survival analyses, the log-rank test was used. Survival probability was plotted over time using Kaplan-Meier survival curves and the trends of survival rates compared between malignancies.

### 2.4. Cox proportional hazards regression analysis was employed to determine the survival rates

To further evaluate the prognostic value of MSLN expression, Cox proportional hazards regression analyses were performed using the survival package in R studio (v4.4.1) [20]. Hazard ratios (HRs) and their respective 95% confidence intervals (CIs) were estimated using both univariate and multivariate Cox regression. The correlation between MSLN expression and overall survival was evaluated using univariate Cox analysis, and the multivariate Cox regression analysis was performed when the clinical data were available, adjusting for other variables as appropriate to help control for confounding, such as age at surgery, sex and pathological stage. To summarize hazard ratios and prognostic associations by cancer type, forest plots and comparative heatmaps have been generated.

### 2.5. Statistical analysis

R studio v.4.4.1 was used for all the analyses. Two-sided non-parametric statistical tests were used where applicable to compare continuous variables. False discovery rate (FDR) correction was performed using the Benjamini-Hochberg method where applicable, and adjusted *p*-values < 0.05 were considered statistically significant. Figures and visualizations have been generated with standard graphical parameters to guarantee consistency and reproducibility between analysis and data sets.

### 2.6. Genetic alteration analysis

Genetic alteration information for MSLN was taken from cBioPortal database (https://www.cbioportal.org/) for Cancer Genomics platform to examine the mutational landscape of MSLN in different cancer types [21]. Tumor-derived TCGA mutation datasets were downloaded and analyzed using R studio (v4.4.1) [22]. The frequency of mutations, the types of mutations and the locations of the mutations in the MSLN gene were analyzed to find out if there are any genomic changes that can be linked to cancer progression and MSLN dysregulation. Multiple classes of genomic alterations such as missense, nonsense, frameshift and splice-site mutations were analyzed. Comparative analyses were also conducted to assess distribution of MSLN mutations in different malignances as well as its possible association with differential MSLN expression patterns. Mutation landscapes and comparison of alteration frequencies were used to visualize mutation distribution and frequencies. Because of the possibility of participation in protein processing and in biological interactions in tumors, mutations situated in the functionally relevant N-terminal region of MSLN were subjected to attention. This analysis was performed to determine if there are any genomic changes in MSLN that could explain the aberrant expression of this gene in MSLN and functional effects in the progression of tumors in all types of cancer. The computational R scripts for mutation and genetic alteration analyses are provided in S3 File.

### 2.7. Functional enrichment and interaction network analysis

Functional enrichment analysis was performed to investigate the biological pathways, molecular functions, and cellular processes associated with Mesothelin (MSLN). A total of 81 interacting genes were retrieved from the BioGRID database (https://thebiogrid.org/) based on experimental evidence, including MSLN interacting genes [23]. All potentially interacting genes were selected to analyze their associated biological function on MSLN. Gene set enrichment analysis (GSEA) was conducted with the web-based functional enrichment analysis tool called Enrichr database (https://maayanlab.cloud/Enrichr/), which combines various biological annotation databases. Gene Ontology (GO) categories, including Biological Process (BP), Molecular Function (MF), and Cellular Component (CC), as well as KEGG pathway enrichment analysis, were performed. Significantly enriched terms were identified based on adjusted *p*-value < 0.05 and combined score ranking as implemented in Enrichr. The complete list of BioGRID-derived MSLN interacting genes is provided in S1 Table.

### 2.8. Drug sensitivity analysis

Drug sensitivity analysis was performed with MSLN expression using pharmacogenomic datasets of Genomics of Drug Sensitivity in Cancer (GDSC) and Cancer Therapeutics Response Portal (CTRP) via the Gene Set Cancer Analysis (GSCA) platform (https://guolab.wchscu.cn/GSCA/#/) [24]. They were developed to use a large-scale anticancer drug screen and a transcriptional profile of cell lines for a systematic review of relationships between gene expression and drug sensitivity. MSLN expression levels were correlated with drug responses in cancer cell lines using Spearman correlation. The *p*-values were adjusted for multiple testing using the false discovery rate (FDR) method where applicable. All available compounds present in the GDSC and CTRP datasets were included in the analysis to avoid selection bias. The effect size and the statistical significance were used to order the results of the correlations.

### 2.9. Structural and pharmacological assessment of MSLN-targeting compounds

#### 2.9.1. Biological rationale for targeting MSLN using small molecules.

Mesothelin (MSLN) is a glycosyl-phosphatidyl-inositol (GPI) anchored cell surface glycoprotein that plays a role in tumor progression, cell adhesion, and communication with the tumor microenvironment (TME) [4]. The best characterized interaction of MSLN is with MUC16 (CA125), which is involved in tumor cell adhesion and dissemination, especially in cancers of the pancreas and ovary [7]. Therefore, targeting the interaction between MSLN and MUC16 has become a promising therapeutic approach to reduce the progression and metastasis of tumors. A number of therapeutic drugs against MSLN are in the clinic, including the use of antibody-drug conjugates (ADCs), immunotoxins, vaccines, chimeric antigen receptor T-cell (CAR-T) therapy and various types of monoclonal antibodies, demonstrating the clinical relevance of MSLN as a therapeutic target associated with cancer [25,26]. But small molecule modulators could provide complementary benefits, such as the ability to target dynamic protein-protein interaction interfaces, decreased manufacturing expenses, improved tissue penetration, and oral bioavailability. As such, structure-based molecular docking, along with pharmacokinetic analysis, prediction of toxicity, and molecular dynamics simulations, were carried out to assess the structural druggability of MSLN and to discover potential small-molecule docking inhibitors that could approach accessible pockets in the protein’s extracellular domain.

#### 2.9.2. Preparation and selection of ligands.

The 3D structures of selected phytochemical compounds were obtained from PubChem database in the Structure Data File (SDF) format. Initially, 31 phytochemical compounds and doxorubicin (control) were selected based on their structural diversity, reported anticancer activity and biological relevance in cancer therapeutics. The preparation of ligands and energy minimization were carried out using pyRx software v.0.8 that includes the AutoDock Vina docking engine [27]. To get energetically stable conformations before docking, energy minimization was performed by employing the Universal Force Field (UFF). The optimized ligand structures were saved in PDBQT format for docking simulation. Based on preliminary docking screening, six compounds (CID: 265237, 10494, 64945, 5280805, and 31703) showing the most favorable binding affinity scores were selected for further structural and pharmacological analyses.

#### 2.9.3. Protein preparation and binding pocket identification.

The 3D structure of the human mesothelin (MSLN) was retrieved from the Protein Data Bank (PDB ID: 7U9J; Chain A) at a 2.09 Å resolution. This structure was selected because of its good structural quality, and suitability for structure-based drug design against the extracellular domain of MSLN. Protein preparations were made with UCSF Chimera (version 1.17.3) [28]. All molecules of water, heteroatoms and nonessential residues were omitted. The polar hydrogen atoms were added and then it was energy minimized to get the optimal geometry for docking studies. Subsequently, AutoDockTools (MGLTools version 1.5.7) was used to assign Gasteiger charges and convert the protein into PDBQT format. The server CASTp 3.0 was used to identify the binding pockets. Pocket 1 was selected from among the predicted cavities owing to its solvent accessible surface area, pocket volume and favorable residue composition. The selected pocket exhibited a solvent-accessible surface area of 1559.517 Å² and a volume of 1376.160 Å³, with a geometric center at X = 13.166, Y = 3.407, Z = 16.168. Important residues included those involved in hydrophobic, electrostatic and hydrogen bond interactions, which contribute to the stability of ligand binding.

#### 2.9.4. Molecular docking analysis.

Molecular docking was carried out using AutoDock Vina algorithm in PyRx software (Version 0.8) [27]. Prior to the docking simulations, both the protein and ligand structures were converted to PDBQT format. The docking grid was centered on Pocket 1 with coordinates X = 13.166, Y = 3.407, Z = 16.168, and grid dimensions of 22 × 22 × 22 Å with a spacing of 1.0 Å. The exhaustiveness of 8.0 was used. The binding affinity was calculated and presented in terms of kcal/mol, and the lowest binding energy pose was selected as the best docked pose. Five top compounds (CID: 265237, 10494, 64945, 5280805, and 31703) were used for redocking to assess docking robustness using Schrödinger Maestro (version 14.3). The Ligand Preparation was done using LigPrep, and Protein Optimization using Protein Preparation Wizard. Glide docking was employed to assess consistency in binding orientation and affinity. Doxorubicin was used as a reference control ligand for benchmarking, being a well-known clinical use broad spectrum anthracycline chemotherapeutic agent and widely used in protein-ligand interaction studies in cancer research.

#### 2.9.5. Interaction analysis and visualization.

Protein-ligand interaction analyses were carried out using BIOVIA Discovery Studio Visualizer (2021) and Schrödinger Maestro (14.3). Ligand binding stability and patterns were analyzed by examining hydrogen bonding, hydrophobic and electrostatic interactions and other non-covalent interactions. To assess the binding mode consistency and the molecular recognition pattern across docking platforms, interactions of residues in the CASTp-derived binding pocket were mapped.

#### 2.9.6. Molecular dynamics simulation and trajectory analysis.

Based on the docking and interaction analyses, the MSLN-Withaferin A complex, which exhibited the most favorable binding profile among the screened compounds, was selected for molecular dynamics (MD) simulation analysis. MD simulations were performed using GROMACS version 2023 to further investigate the structural stability and dynamic behavior of the complex [29]. CHARMM36 force field was used for the generation of topology for proteins and parameters for ligands were generated using a CHARMM General Force Field (CGenFF). Solvation (water) was performed for the protein-ligand complex using the TIP3P water model, inside a cubic simulation box. System was neutralized with counter ions (Na⁺ and Cl^-^) to simulate physiological ionic conditions. To remove the steric clashes and unfavorable molecular interactions, energy minimization was done by steepest descent algorithm. After minimization the equilibration was performed under both NVT and NPT ensembles at 300K and 1 bar pressure, respectively by employing V-rescale thermostat and Parrinello-Rahman barostat. To stabilize the protein-ligand complex, position restraints were kept during equilibration. About 100 ns production MD simulation was then carried out using a 2-fs integration timestep and with periodic boundary conditions. The long-range electrostatic interactions have been calculated with the Particle Mesh Ewald (PME) method, and covalent interactions of bonds including hydrogen atoms were constrained with the LINCS algorithm. The simulation time was set as 100 ns, which seemed adequate in the present case to judge the stability and conformational changes of the protein-ligand complex. Trajectories analyses were carried out to evaluate the stability of the structure and the conformational dynamics of the MSLN-Withaferin A complex over the simulation time. Root mean square deviation (RMSD), root mean square fluctuation (RMSF), radius of gyration (Rg) and hydrogen bond analysis were performed. All donors and acceptors with distances less than or equal to 0.35 nm and angles smaller than or equal to 30° were found as potential hydrogen bonds. Dominant collective motions of the complex were identified using covariance matrices, generated from Cα atomic fluctuations obtained from the simulation trajectories by principal component analysis (PCA). To further quantify the binding affinity of the MSLN-Withaferin A complex, Molecular Mechanics/Poisson-Boltzmann Surface Area (MM/PBSA) calculations were performed using snapshots extracted from the MD trajectory. The binding free energy (ΔGbind) was calculated as the sum of van der Waals, electrostatic, polar solvation and non-polar solvation energy components. Moreover, energy decomposition analysis per residue was performed to provide a picture of the main amino acid residues involved in ligand binding and in complex stabilization.

#### 2.9.7. ADMET, drug likeness, toxicity prediction.

SwissADME and ProTox-III web servers were used for assessment of the pharmacokinetic profile, drug likeness and toxicity profile of top-ranked ligand, Withaferin A (CID: 265237) [29]. Drug-likeness assessment including Lipinski’s rule of five and physicochemical properties, gastrointestinal absorption, water solubility, H- bond donor and acceptor, etc. was conducted using SwissADME (https://www.swissadme.ch/). These analyses were carried out to appreciate its oral drug potential and the pharmacokinetic behavior at least in mice and rats. The ProTox-III (https://tox.charite.de/protox3/) platform was used for prediction of toxicological properties. Acute oral toxicity (LD50), toxicity class prediction, hepatotoxicity, cardiotoxicity, respiratory toxicity, immunotoxicity, carcinogenicity, mutagenicity and cytotoxicity endpoints were evaluated for toxicity assessment. The relative safety profile of Withaferin A was estimated to be using the predicted LD50 value and toxicity class. These analyses were carried out to determine the pharmacological feasibility, safety data, and possible constraints of the use of Withaferin A as a potential MSLN-targeting compound, before experimental validation.

#### 2.9.8. Ethical statement.

This study was conducted exclusively using publicly available datasets and computational analyses and did not involve direct human participants, animal experiments, or clinical samples. Therefore, ethical approval and informed consent were not required.

## 3. Results

### 3.1. MSLN is overexpressed across multiple human cancers

**Fig 1:**
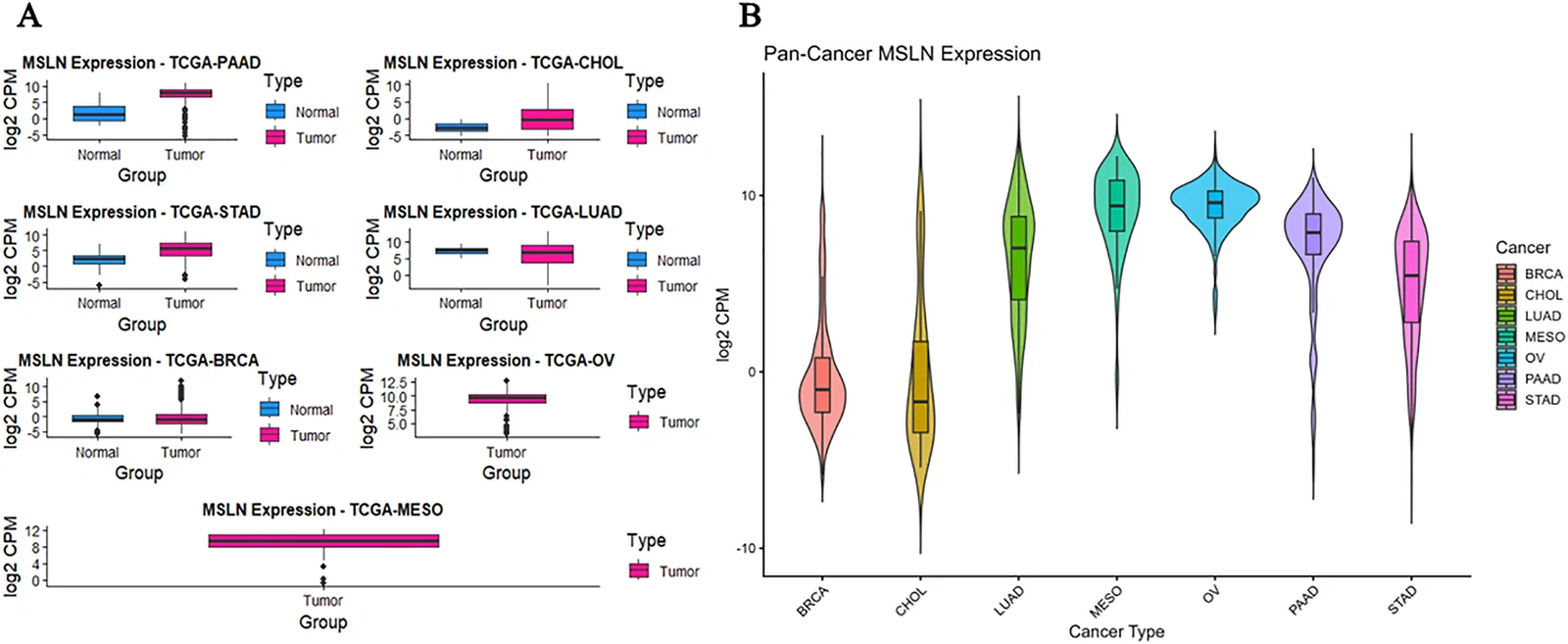
MSLN expressions in various human cancers. (A) Differential expressions of MSLN in tumor vs matched normal tissues of PAAD, CHOL, STAD, LUAD and BRCA. All expression values are shown as log2 CPM with the Wilcoxon rank-sum test for statistical significance. (B) Comparison of MSLN expression levels among BRCA, CHOL, LUAD, MESO, OV, PAAD, and STAD tumors. Violin plots show the mean expression level and the spread of variance of MSLN expression within each type of cancer.

The expression pattern of mesothelin (MSLN) was assessed across seven different malignancies: pancreatic adenocarcinoma (PAAD), cholangiocarcinoma (CHOL), stomach adenocarcinoma (STAD), lung adenocarcinoma (LUAD), breast invasive carcinoma (BRCA), ovarian serous cystadenocarcinoma (OV) and mesothelioma (MESO). Differential expression analysis showed that MSLN expression was significantly higher in tumor samples compared to normal samples in PAAD, CHOL, STAD, LUAD and BRCA (Wilcoxon rank-sum test, *p* < 0.05; Fig 1a) S2 Table. Within this group, the level of MSLN overexpression, compared with normal tissues, was one of the highest for PAAD. In cases of OV and MESO, no normal tissue samples were available in TCGA and as such only the expression level of the tumor associated samples were assessed. The expression of MSLN was found to be remarkably high for both cancer types, which is in the known characteristic expression pattern of MSLN as a biomarker in ovarian cancer and mesothelioma. The results also provide additional evidence of the role of MSLN in tumor representation and growth. To compare the MSLN expressions across all selected cancer types, a pan-cancer expression analysis was performed using tumor samples (Fig 1b). MSLN expression exhibited significant intertumoral variability. The top three median expression levels were achieved for MESO, OV, LUAD and PAAD while BRCA and CHOL showed relatively lower expression levels. However, low levels of MSLN were detected in all cancer types, which indicated that there is a heterogeneous wide-spread dysregulation of MSLN in human cancers. In summary, these results suggest that MSLN is consistently over expressed in clinically important malignancies, especially PAAD, OV, and MESO and may be useful for both diagnosis and therapy.

### 3.2. Independent GEO cohorts validate the overexpression and diagnostic performance of MSLN

**Fig 2:**
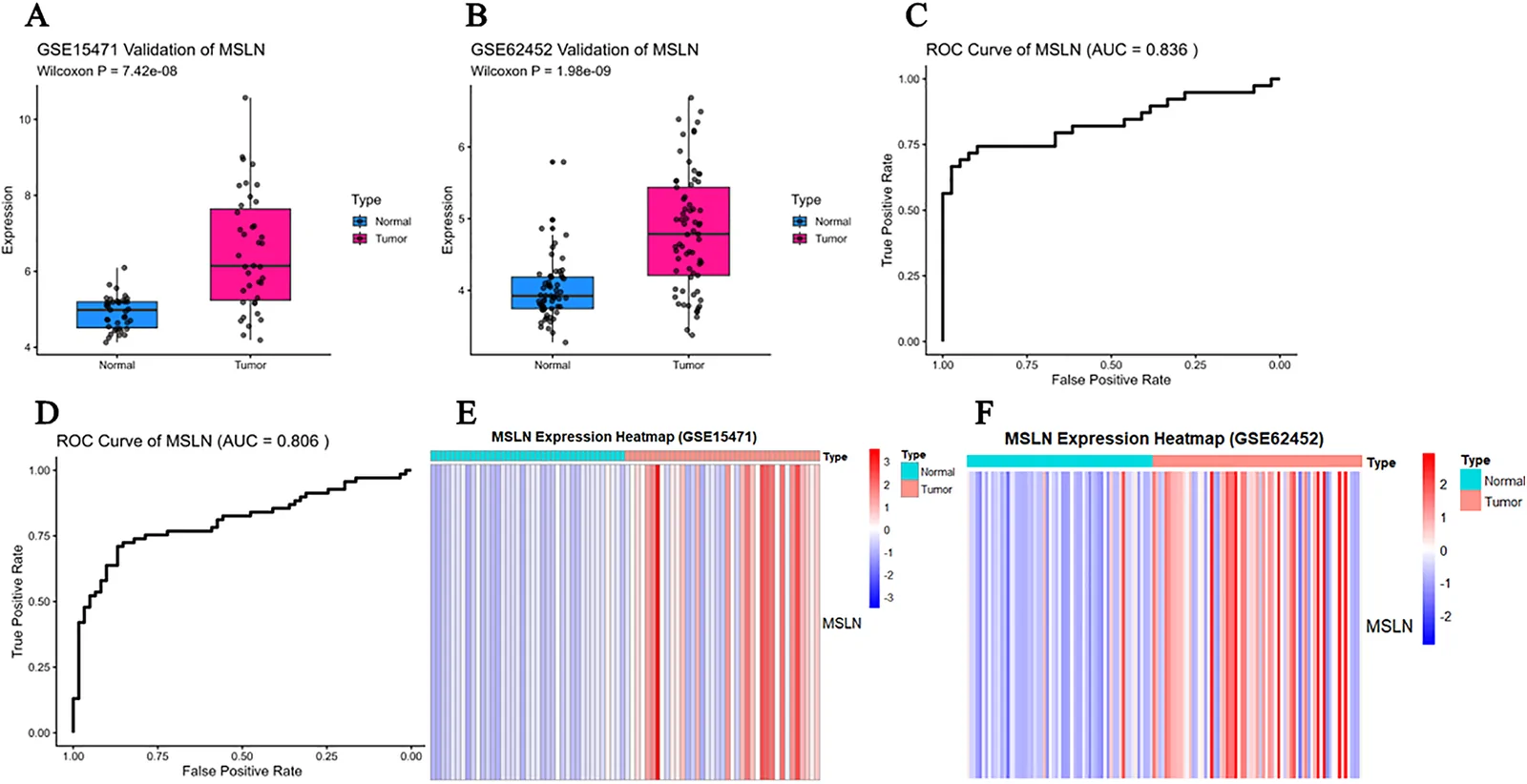
Independent validation of MSLN expression and diagnostic performance in pancreatic adenocarcinoma with GEO datasets. The differential expression of MSLN of normal versus tumor tissues in GSE15471 (A) and GSE62452 (B) are shown respectively. The Wilcoxon rank-sum tests were used for statistical significance. ROC curve analyses assessing the diagnostic value of MSLN for pancreatic tumor tissues versus normal tissues in the GSE15471 (C) and GSE62452 (D). AUC values are listed in each panel. Heatmap representation of MSLN expressions in normal and tumor samples from GSE15471 (E) and GSE62452 (F). The red coloration shows high levels of expression, and the blue coloration shows low levels of expression.

MSLN expression was assessed in two independent GEO datasets (GSE15471 and GSE62452) to validate the findings derived from the TCGA. The expression of MSLN was significantly upregulated in the pancreatic tumor tissue samples compared to the normal pancreatic tissue samples in both validation sets (Wilcoxon test; *p* = 7.42 × 10^−8^ for GSE15471, and *p* = 1.98 × 10^−9^ for GSE62452; Figs 2a and 2b) and S3 and S4 Tables. Receiver operating characteristic (ROC) curve analyses were used to evaluate the diagnostic value of MSLN. MSLN was also shown to have a reasonably good discriminatory ability between tumor and normal samples, with an AUC of 0.836 (GSE15471) and 0.806 (GSE62452) (Figs 2c and 2d). The results show that MSLN expression can accurately discriminate cancers and normal tissues of the pancreas in several independent cohorts of patients. In addition, the expression of the MSLN gene in the validation datasets was used to generate heatmap between tumor and normal samples, showing a good separation between them (Figs 2e and 2f). The expression levels in the tumor samples were mostly high while expression levels in normal samples were comparatively low. The observed clustering pattern also further reinforces the results of MSLN overexpression that are robust and reproducible in pancreatic adenocarcinoma. Together, the highly similar expression differences, good diagnostic value, and robust diagnostic gene expression patterns in each of the individual GEO cohorts support the reliability of the TCGA findings and further confirms the potential of MSLN as a diagnostic marker in pancreatic adenocarcinoma.

### 3.3. ROC analysis identifies PAAD and STAD as cancers with the highest diagnostic potential of MSLN

**Fig 3:**
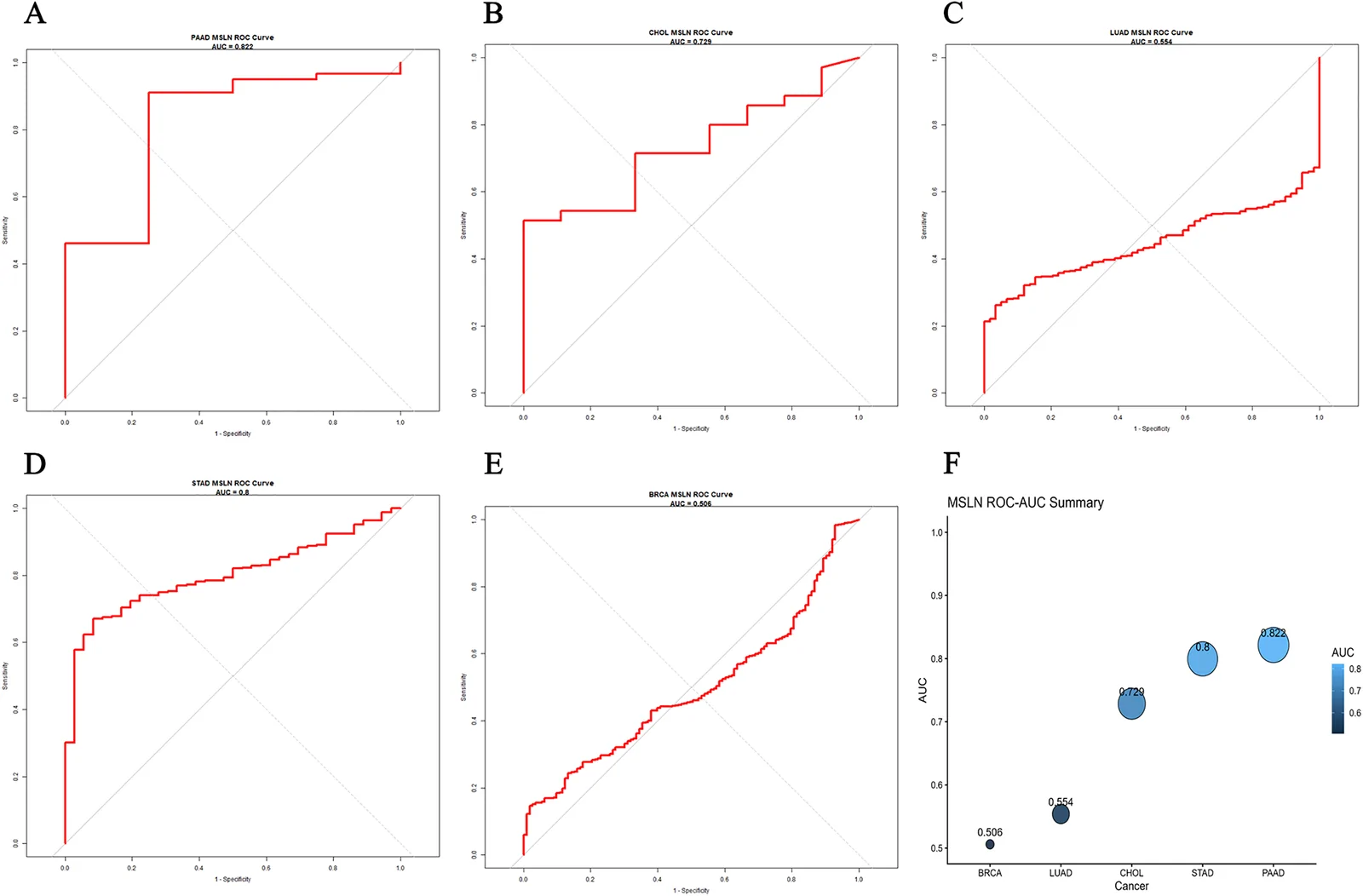
Expression of MSLN in various types of cancer and its diagnostic value. Receiver operating characteristic (ROC) curves to demonstrate discriminative performance of MSLN expression between the different tumor tissue and normal tissue in (A) PAAD, (B) CHOL, (C) STAD (D) LUAD and (E) BRCA. The bubble plot (F) shows AUC values for all cancer types analyzed and reveals that MSLN generally has a good diagnostic performance in PAAD and STAD.

Receiver operating characteristic (ROC) curve analysis was used to assess the diagnostic value of MSLN expression across the cancer of interest. The AUC value of MSLN was demonstrated the highest of all at 0.822 in pancreatic adenocarcinoma (PAAD), followed by stomach adenocarcinoma (STAD) with an AUC value of 0.800, demonstrating good discrimination between tumor and normal tissues (Fig 3 and Table 1). The diagnostic accuracy of cholangiocarcinoma (CHOL) was moderate (AUC: 0.729) compared with lung adenocarcinoma (LUAD) (AUC: 0.554). Likewise, breast invasive carcinoma (BRCA) had low discriminatory ability with an AUC value of 0.506, a near random classification. Overall, these results indicate that the diagnostic value of MSLN will be more significant in PAAD and CHOL rather than depending solely on it in LUAD and BRCA. The AUC values and ROC curves for MESO and OV cancers weren’t generated, because these datasets didn’t contain normal samples.

**Table 1:** AUC values of MSLN in the respective cancers.

Cancer	AUC (95% CI)
PAAD	0.821
CHOL	0.728
STAD	0.799
LUAD	0.554
BRCA	0.506

### 3.4. MSLN is positively associated with tumor progression in PAAD

**Fig 4:**
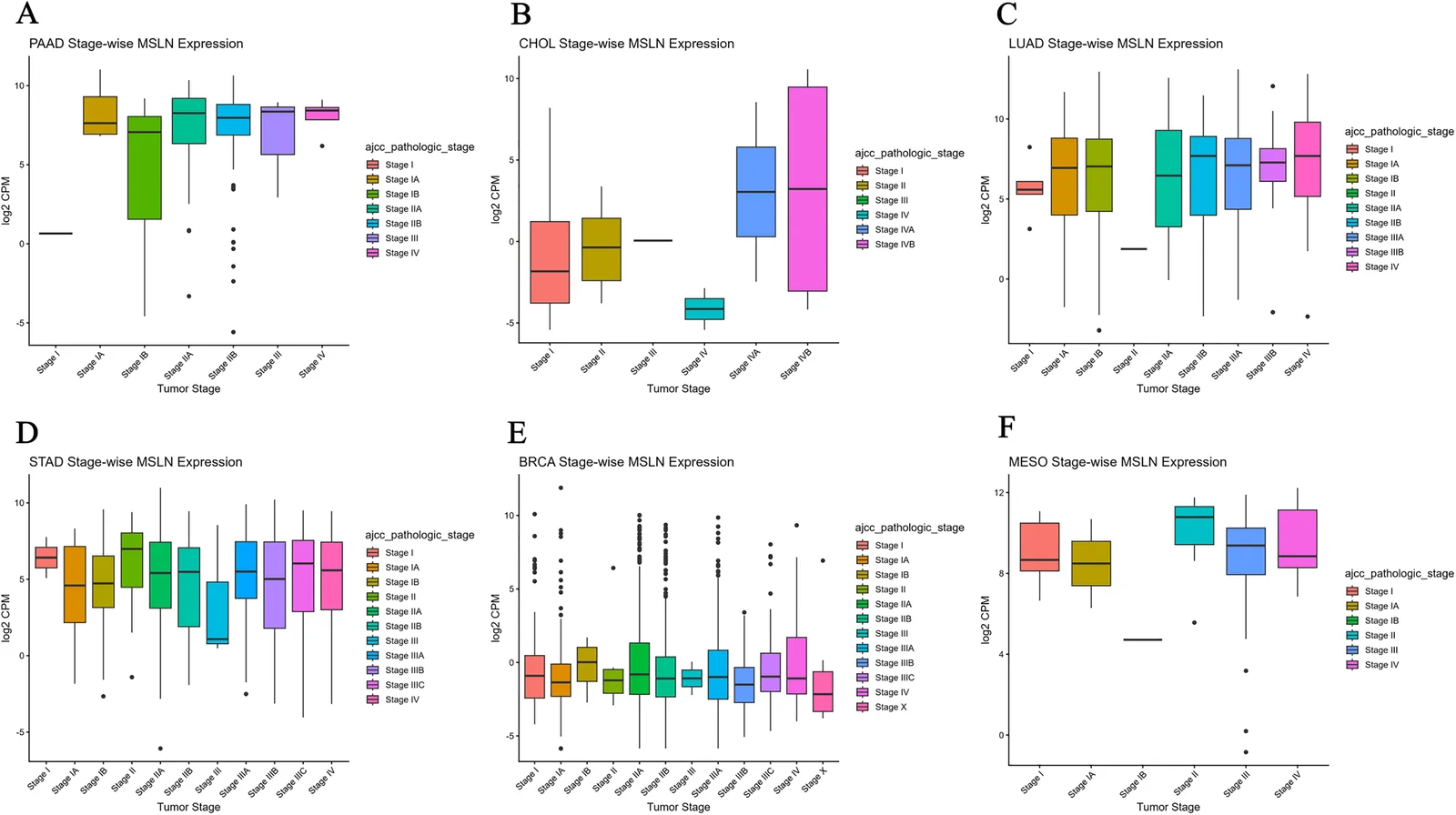
Differential expression of MSLN at mRNA levels in different stages of selected cancer types. Boxplots illustrate MSLN expression levels (log2 CPM) stratified by pathological tumor stage in (A) PAAD, (B) CHOL, (C) LUAD, (D) STAD, (E) BRCA, and (F) MESO. There were consistently high levels of MSLN expression in all disease stages of PAAD and MESO, while the expression from other diseases (CHOL, LUAD, STAD and BRCA) was more variable, showing different stage-dependent expressions. The results indicate that the correlation between MSLN expression and tumor progression may be different in different types of cancer.

To explore the correlation between the expression of MSLN with tumor progression, the expression of MSLN was analyzed in PAAD, CHOL, LUAD, STAD, BRCA, and MESO cohorts in stage-wise manners (Fig 4). MSLN expression was found to be constantly high in both PAAD and MESO throughout pathological stages, indicating that MSLN expression is consistently kept high during disease evolution in these cancers. PAAD showed relatively higher expression in more advanced stages, suggesting that there may be a correlation between the expression of MSLN and tumor progression. Likewise, MESO demonstrated consistently high levels of MSLN expression throughout all stages, consistent with its reputation as a malignancy that is rich in MSLN. Conversely, the expression level of MSLN was more variable in the other categories (CHOL, LUAD, STAD and BRCA) without an apparent monotonic stage-dependent increase or decrease. Within each stage changes were found but these did not necessarily indicate upregulation as disease stage advanced. Together, these results suggest that MSLN expression can vary between cancer types about a correlation with tumor stage, most clearly in PAAD and MESO. The stage plot for OV cancer could not be generated due to the unavailability or insufficient clinical stage information in the dataset.

### 3.5. MSLN expression shows cancer-type-specific associations with overall survival

**Fig 5:**
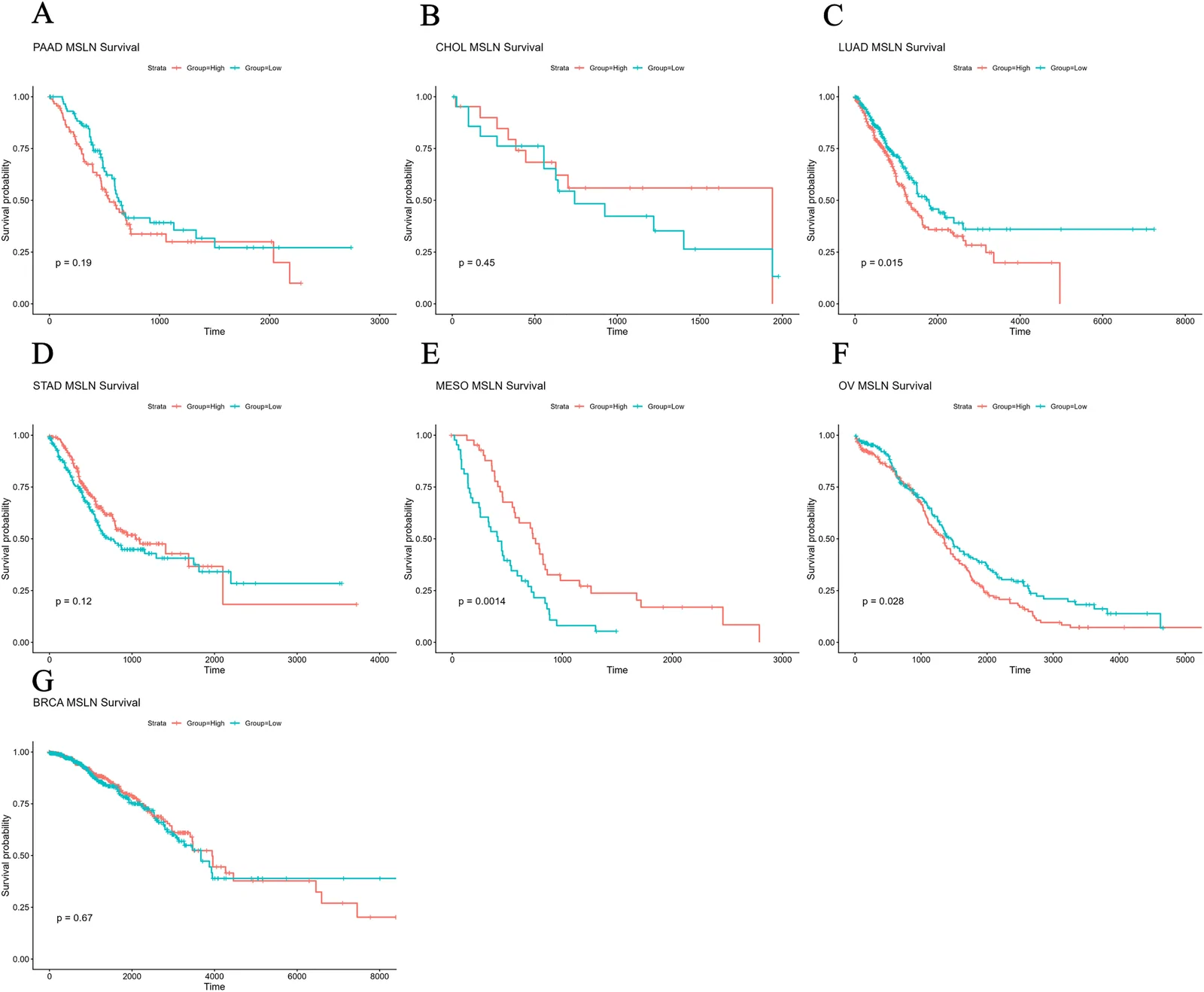
Kaplan-Meier overall survival analysis according to MSLN expression levels across multiple cancer types. The patients were divided into groups of high and low MSLN expressions based on median expression level. High MSLN expression was significantly associated with poor overall survival in LUAD (*p* = 0.015), MESO (*p* = 0.0014), and OV (*p* = 0.028); however, there was no statistically significant difference in overall survival in PAAD (*p* = 0.19), CHOL (*p* = 0.45), STAD (*p* = 0.12), and BRCA (*p* = 0.67). Logrank test was used to assess statistical significance.

Kaplan-Meier survival analysis revealed significant associations between elevated MSLN expression and poorer overall survival in LUAD, MESO, and OV patients (Fig 5). However, no survival differences were found among PAAD, CHOL, STAD, and BRCA cohorts. These findings suggest that the prognostic relevance of MSLN varies among cancer types. Notably, Kaplan-Meier analysis did not reveal a significant survival difference in PAAD. Cox proportional hazards analysis identified MSLN expression as a significant prognostic factor. The difference might reflect methodological differences, because Cox regression measures the continuous effect of gene expression, while the Kaplan-Meier was based on dividing gene expression into groups, which may reduce statistical power.

### 3.6. Cox regression revealed cancer-type-specific prognostic associations of MSLN

**Fig 6:**
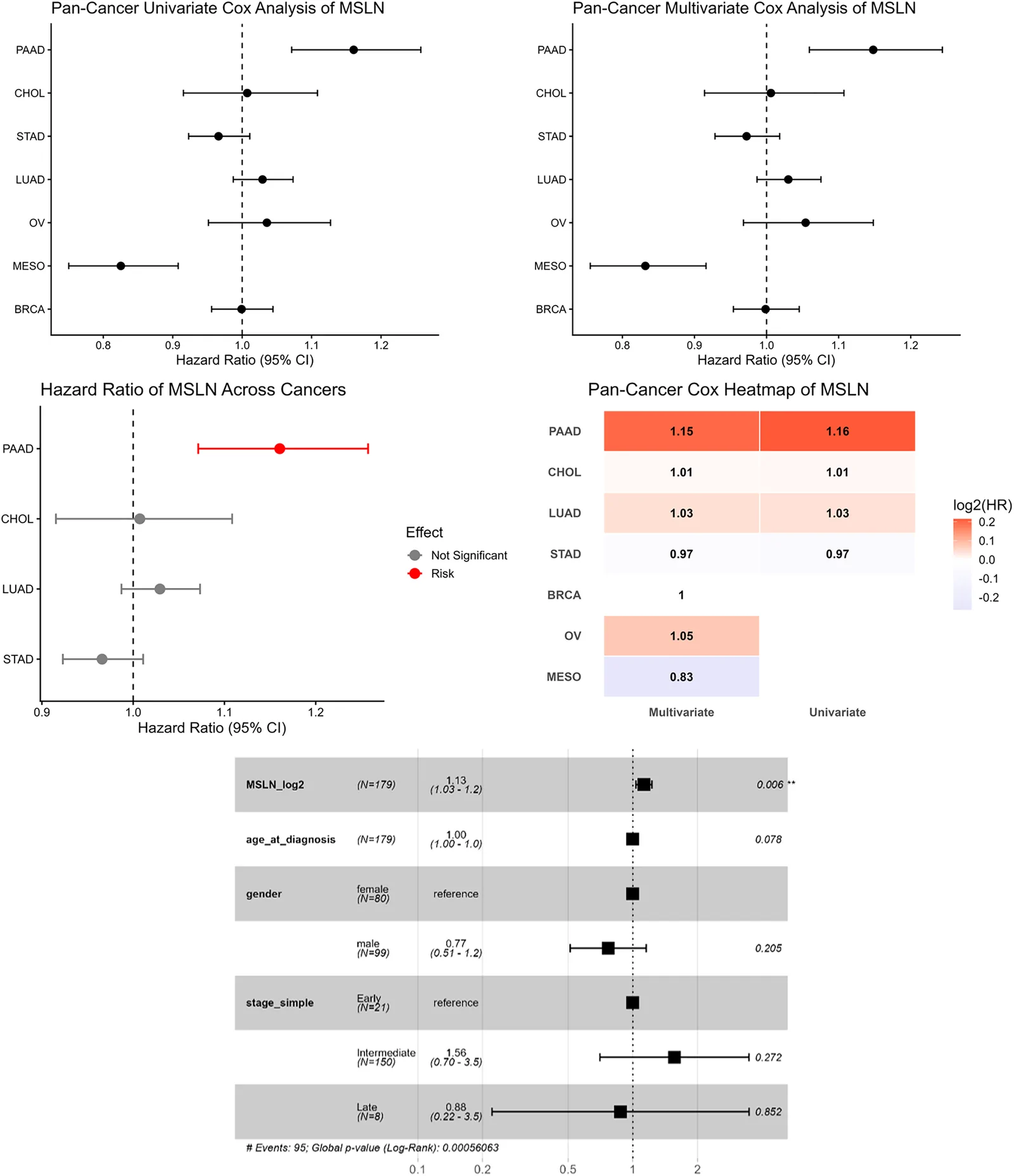
MSLN Cox proportional hazards analysis in various cancer types. Univariate and multivariate Cox regression analyses were performed to evaluate the prognostic significance of MSLN expression. Hazard ratios (HRs) and 95% confidence intervals are shown for forest plots and summarized within the heatmap for estimates of HRs for all types of cancer. Increased mortality risk in PAAD was significantly associated with increased expression of MSLN but these correlations were weak (or insignificant) in the other cancer types.

Univariate and multivariate Cox proportional hazards analyses were further conducted across MSLN-high cancers to further assess the prognostic significance of MSLN. Higher expression levels of MSLN were a significant independent predictor in PAAD (Multivariate HR = 1.14 (95% CI = 1.05–1.24, *p* = 0.0007)) but not in CHOL, LUAD, OV, MESO and BRCA (Fig 6 and S5 Table). Interestingly, the Kaplan-Meier analysis did not show the difference in survival rate to be statistically significant in PAAD, whereas the Cox regression analysis revealed MSLN to be statistically significant, which reflects the increased statistical power of the analysis with continuous variables, and inclusion of clinical covariates.

### 3.7. MSLN mutations have little frequency and are mostly located in the N-terminal region

**Fig 7:**
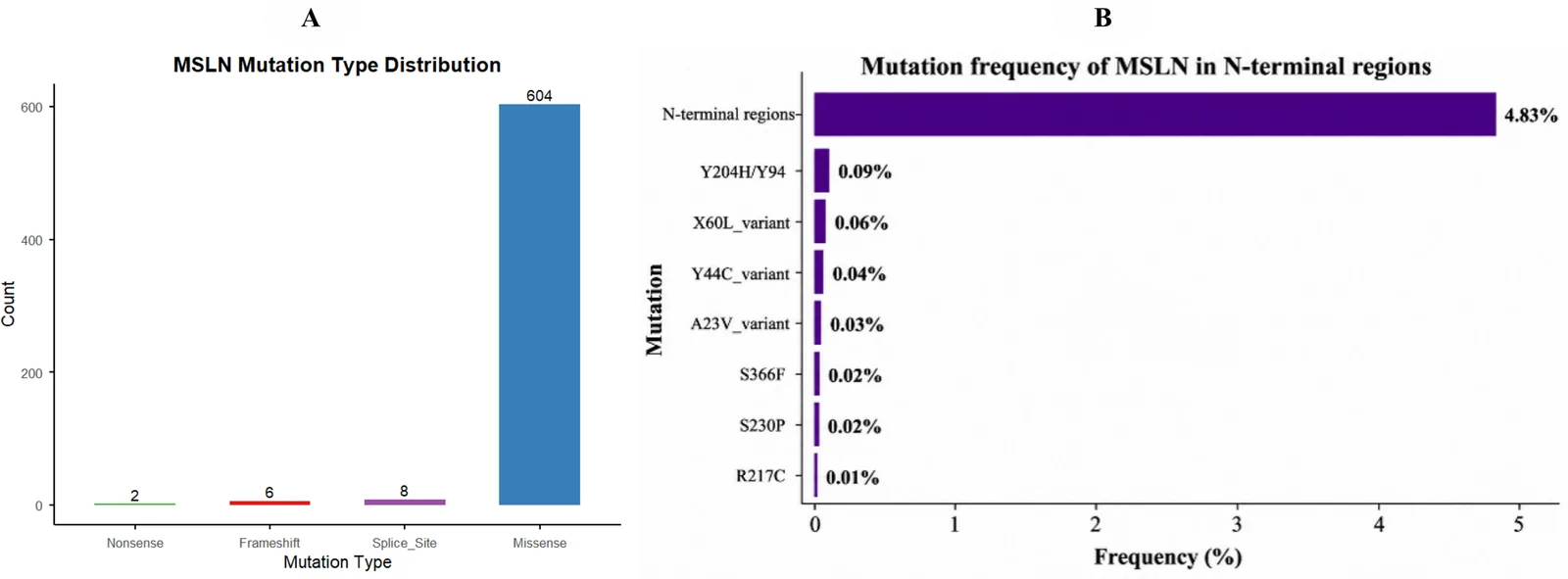
Genetic alterations of MSLN. (A) Frequency of classes of mutation, with missense mutations accounting for most of the different types of mutation, (B) Frequency of recurrently occurring mutations found in the N-terminal region of MSLN, showing the overall very low mutation frequency of the gene in various kinds of cancer.

To assess the role of genomic changes in the dysregulated expression of MSLN in cancer, the mutational spectrum of MSLN was investigated in the tumor sets analyzed. Overall, genetic alterations in MSLN were relatively uncommon. Missense mutations were the most common class of mutations detected with low numbers of frameshift, nonsense and splice-site mutations (Fig 7a). Further analysis of the recurrent variants indicated that most of them were confined in the N-end portion of MSLN as shown in Fig 7b. The N-terminal domain (NTD) was the most affected site with overall mutation frequencies being low (<5%). A total of six other variants (Y204H/Y194, X60L, Y44C, A23V, S366F, S230P and R217C) were found at much lower frequencies. The high percentage of low-frequency mutations indicates that genetic mutations are not likely the major cause of MSLN over-expression for the cancers analyzed. The overexpression seen is especially prominent in pancreatic adenocarcinoma and might rather reflect transcription and epigenetic regulation than frequent coding alterations. However, the clustering of changes in the N-terminal region could suggest relevance to function and be examined further in experiments.

### 3.8. Functional enrichment analysis identifies pathways related to immune regulation and oncogenic signaling pathways associated with MSLN interaction network

**Fig 8:**
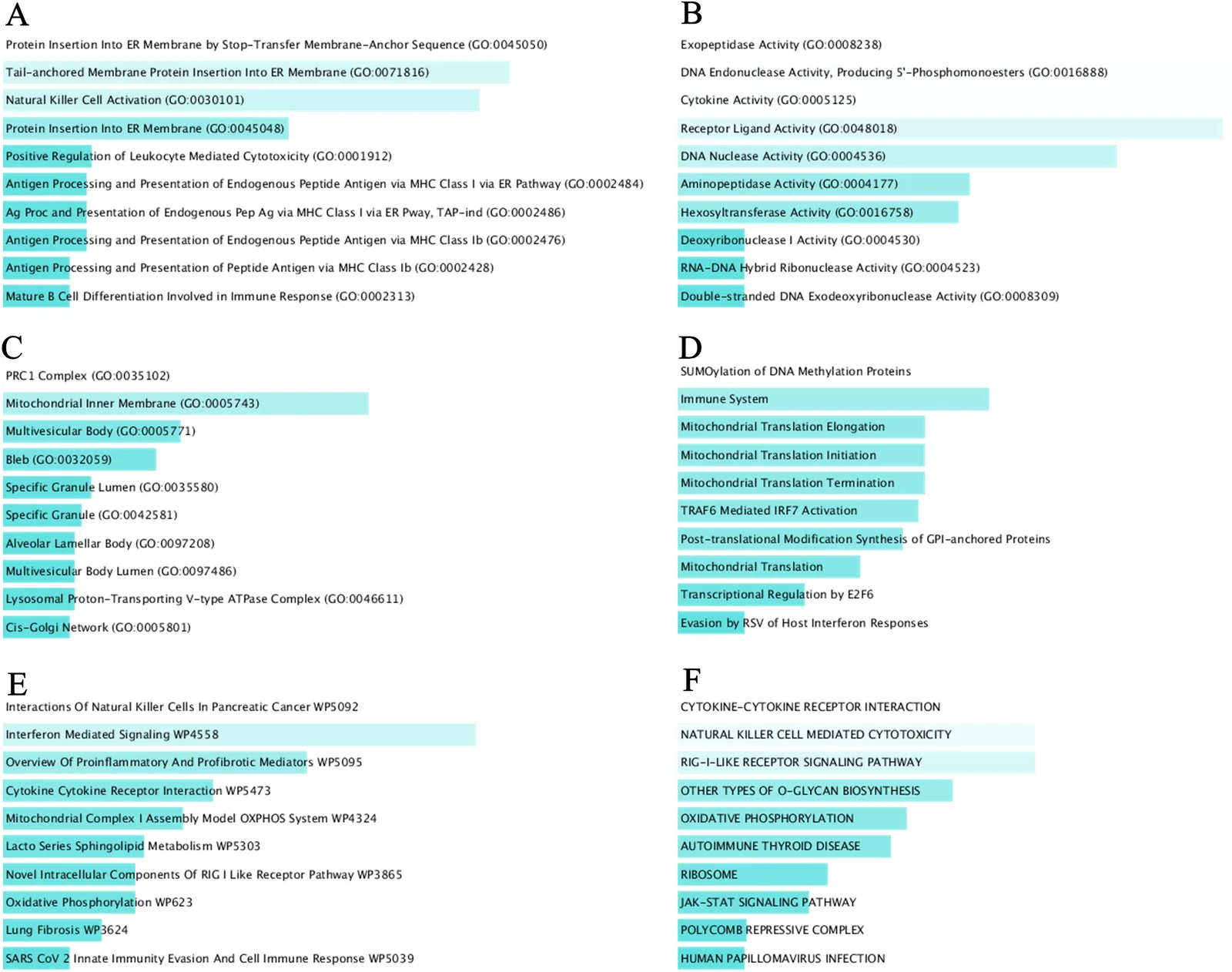
Enrichment analysis of the MSLN-interacted genes. (A) Biological processes, (B) Molecular functions, (C) Cellular components, (D) Reactome pathways, (E) Wiki pathways, (F) KEGG pathways.

Functional enrichment analysis was conducted to determine the biological functions of Mesothelin (MSLN) in its protein interaction network. A total of 82 experimentally validated MSLN-associated interacting genes were retrieved from the BioGRID database and subjected to enrichment analysis using Enrichr. This method enabled a fair estimation of the full set of biological roles linked to the entire MSLN interaction network. Biological Process analysis using Gene Ontology (GO) revealed that immune related and tumor associated processes were significantly enriched, including those related to immune response regulation, leukocyte activation, cytokine mediated signaling and cell adhesion (Fig 8a). GO molecular function analysis revealed high enrichment of receptor binding, protein-protein interaction activity, transcriptional regulation, and nucleic acid binding activities, including functional diversity of the MSLN interaction network (Fig 8b). GO cellular component analysis also showed that membrane associated complexes, extracellular regions and organelle related structures were enriched, which is consistent with the membrane bound nature of MSLN (Fig 8c). Reactome, Wiki and KEGG pathways enrichment analyses revealed that several pathways significantly involved in carcinogenesis or in the immune system, such as cytokine–cytokine receptor interaction, JAK-STAT signaling pathway, natural killer cell-mediated cytotoxicity, and other oncogenic pathways, were amongst the pathways represented by significantly enriched genes related to MSLN (Figs 8d-f). Overall, these data indicate that MSLN is complexly regulated in immune-regulation, cell communication and tumor progression. The list of all statistically significant (adjusted *p*-value < 0.05) enriched GO terms and KEGG pathways is given in S6–S11 Tables.

### 3.9. MSLN expression is associated with drug response across GDSC, and CTRP datasets

**Fig 9:**
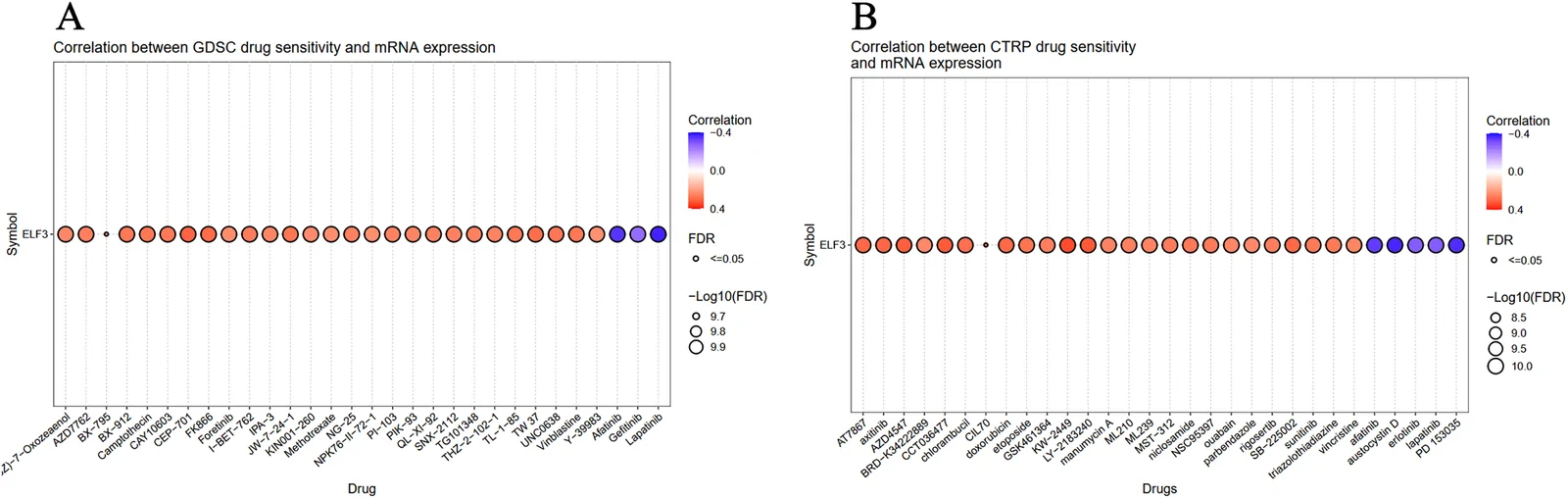
Drug sensitivity analyses of the MSLN. (A) GDSC drug, (B) CTRP drug.

This study analyzed the association between MSLN expression and drug sensitivity using GDSC and CTRP datasets (Fig 9 and S12 and S13 Tables). In general, MSLN exhibited a wide range of associations with drug class. The major association of high MSLN expression was with resistance to various anti-cancer drugs. For both datasets, positive significant correlation was found for both cell cycle inhibitors raised as indicated (BI-2536, GSK461364, vincristine) and PI3K/AKT/mTOR inhibitors such as PI-103, AZD8055 and OSI-027. Such doxorubicin, etoposide, and topotecan, also presented positive correlation, suggesting lower sensitivity in MSLN-high conditions. Conversely, MSLN-high expression correlated with higher drug sensitivity to inhibitors of the EGFR and the MAPK pathway, such as erlotinib, afatinib, trametinib, selumetinib and PD-0325901. There were also negative correlations with dasatinib and saracatinib. Overall, both datasets reflected similar trends, indicating a correlation between MSLN and global chemoresistance and a specific sensitivity to EGFR/MAPK-targeting drugs.

### 3.10. Analysis of molecular docking and interaction

**Fig 10:**
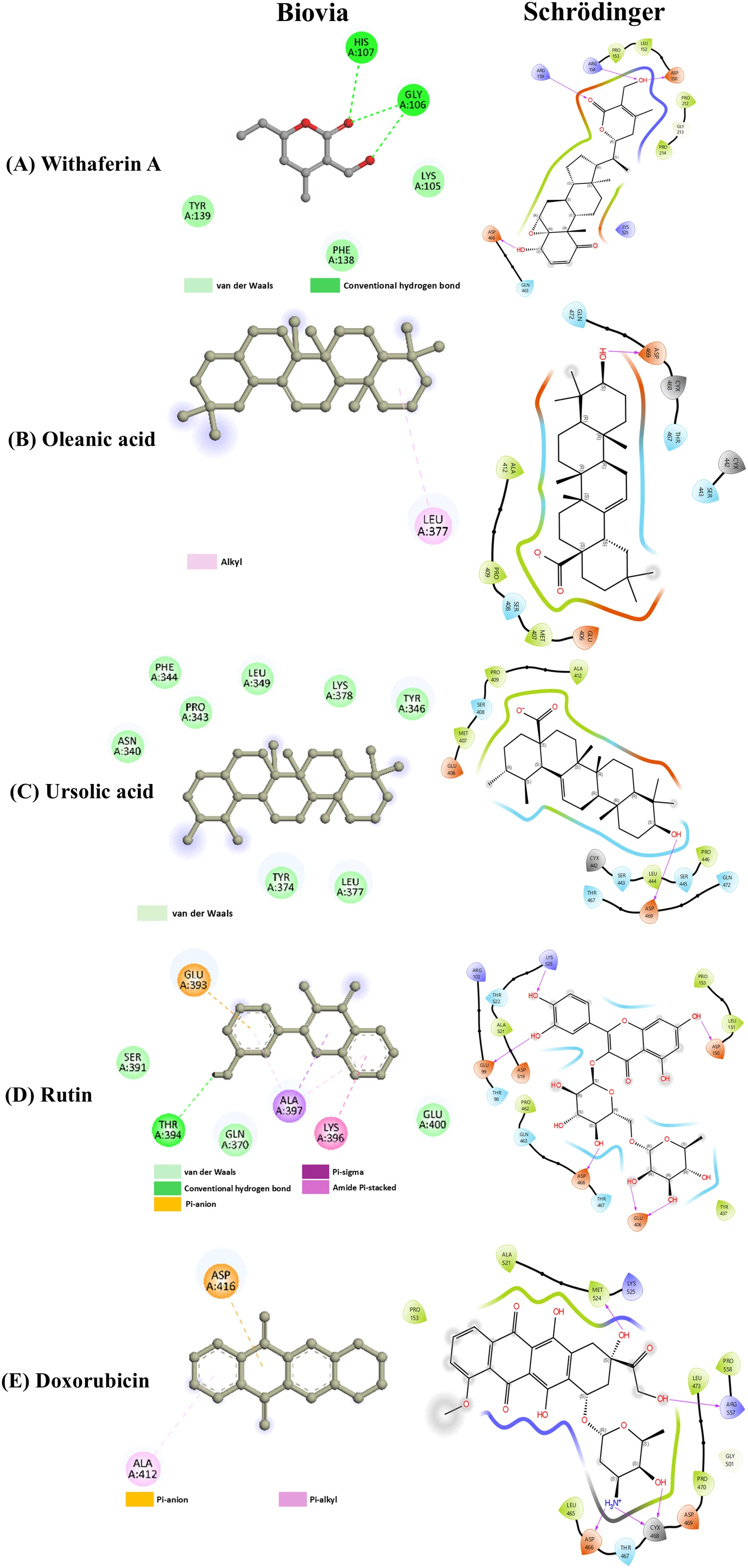
Interactions and binding poses of MSLN with selected drug like molecules. (A) withaferin A, (B) oleanic acid, (C) ursolic acid, (D) rutin, and (E) doxorubicin.

Before molecular docking, the binding pocket of MSLN was predicted, and the capability of the binding pocket to accommodate the ligand was evaluated. The chosen binding cavity displayed a variety of amino acid residues such as Lys, Asp, Glu, His, Tyr, Ser, Thr, Asn, Gln and Cys residues. The presence of charged residues, polar and hydrophobic residues suggests that the pocket can accommodate ligand binding via multiple modes of interaction such as hydrogen bonding, electrostatic, and hydrophobic contacts. Molecular docking was conducted using PyRx (AutoDock Vina) for the assessment of therapeutic potential of selected phytochemicals against MSLN and further supported by molecular docking validation using Maestro Glide software. Doxorubicin as a reference anti-cancer drug was used for comparative analysis. Withaferin A showed the highest binding affinity (−8.0 kcal/mol) with MSLN as compared to all screened compounds in PyRx. Oleanic acid, Ursolic acid and Rutin also exhibited promising binding ability with binding affinity equal to −7.7, −7.6 and −7.5 kcal/mol while Doxorubicin had comparatively lesser binding affinity of −7.1 kcal/mol (Table 2). To confirm these results, docking was carried out again on Maestro Glide program. Consistent with the PyRx results, Withaferin A remained the top-ranked ligand with a Glide score of −7.3 kcal/mol. Doxorubicin exhibited the second-highest Glide score (−6.4 kcal/mol), while Rutin, Oleanic acid, and Ursolic acid showed lower scores ranging from −5.1 to −5.5 kcal/mol (Table 2). It is confirmed by the docking result between the two platforms that the predicted ligand-MSLN interactions are reliable. The interaction analysis revealed that Withaferin A participated in hydrogen-bond interaction with HIS107 and GLY106 in the predicted binding cavity of MSLN (Fig 10a). Other hydrophobic interactions take place between the remaining residues (LYS105, PHE138 and TYR139) and the protein, supporting the ligand-protein complex. The combination of favorable binding energy and multiple stabilizing contacts suggests a strong interaction between Withaferin A and MSLN. Oleanic acid had a binding affinity of −7.7 kcal/mol with the main contacts around the binding pocket being hydrophobic contacts (Fig 10b). Limited polar interactions were observed, indicating that hydrophobic forces may contribute substantially to stabilization of the complex. Likewise, Ursolic acid also showed good binding with MSLN having a docking score of −7.6 (Fig 10c). The residues LEU377, LEU349, TYR374, PRO348 and ASN340 mainly supported the ligand by hydrophobic interactions, indicating that van der Waals interactions are important to maintain the docked conformation. The interaction profile of rutin was found to be distinct with multiple hydrogen-bond and polar interactions (Fig 10d). Residues THR394 and SER391 engaged in hydrogen bonding and ALA397 and LYS396 had other stabilizing contacts. Although it has a lot of interaction sites, it still had poorer binding affinity as compared with Withaferin A. Reference compound Doxorubicin was in hydrogen-bond and hydrophobic contacts with residues ASP416 and ALA412 (Fig 10e). Doxorubicin had measurable binding towards MSLN, but its docking score was less than that of Withaferin A, suggesting that the binding was weak in the pocket compared to Withaferin A. Collectively, the docking and interaction analyses identified Withaferin A as the most promising MSLN-binding compound. The binding affinities of all complexes with phytochemicals classes are provided in S14 Table.

**Table 2:** Binding affinities of the top 4 ligands and control with MSLN by Pyrx.

		Docking by Pyrx	Glide docking	
CID	Ligands	Binding Affinity (kcal/mol)	Binding Affinity (kcal/mol)	Glide emodel
265237	Withaferin A	−8.0	−7.3	−73.6
10494	Oleanic acid	−7.7	−5.3	−55.4
64945	Ursolic acid	−7.6	−5.1	−55.1
5280805	Rutin	−7.5	−5.5	−55.5
31703 (Control)	Doxorubicin	−7.1	−6.4	−64.4

### 3.11. Structural stability analysis by molecular dynamics simulation

**Fig 11:**
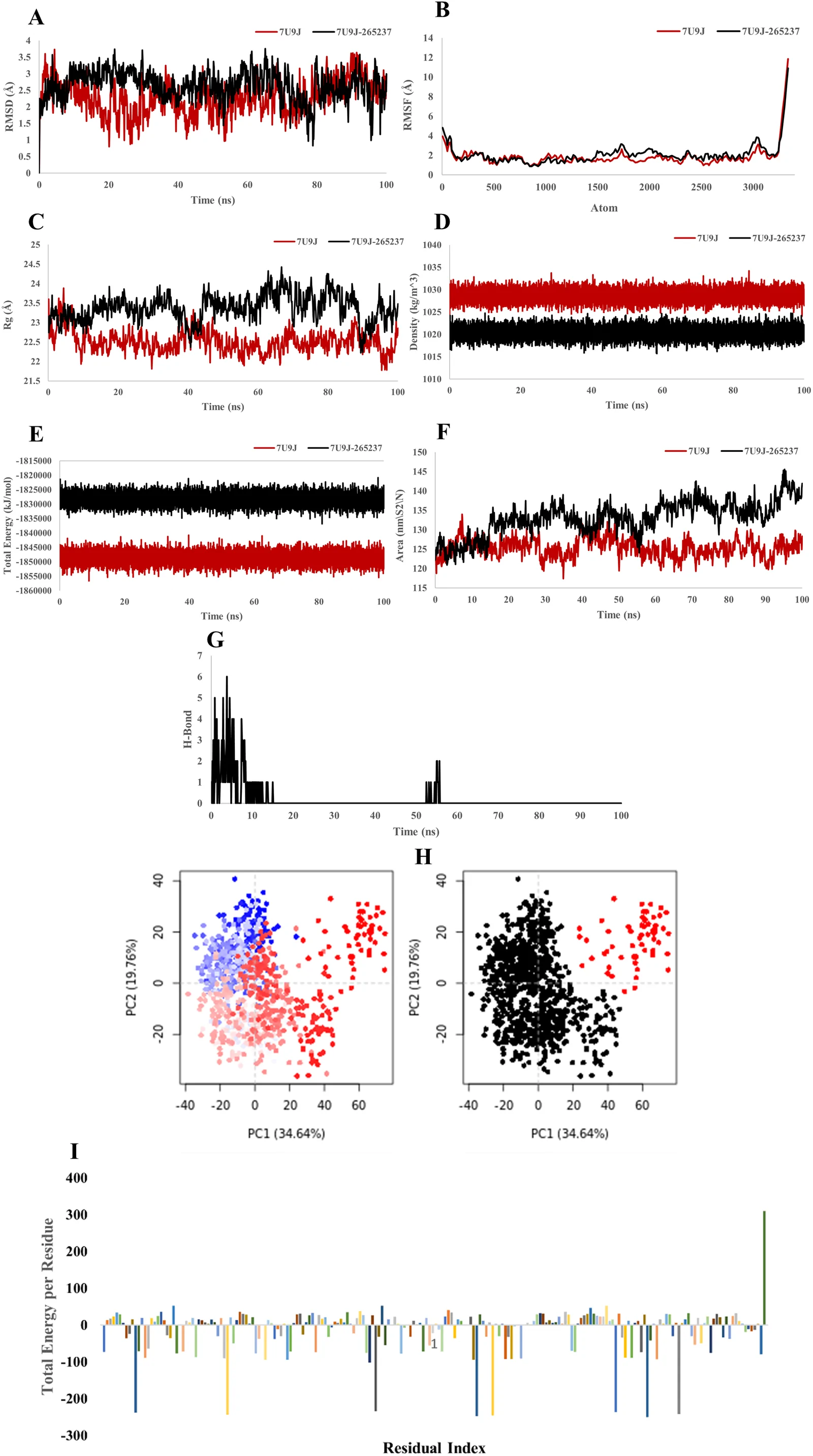
The structural stability analysis of the MSLN and MSLN-Withaferin A complex by molecular dynamics simulation analysis. (A) The root mean square deviation (RMSD) curves of both apo protein (7U9J) and the apo-complex with a given ligand in a 100 ns simulation, (B) RMSF profile of flexibilities on the residue level over the course of the simulation, (C) Rg plot is the total compactness of the protein structure during the simulation. The comparatively constant values show that the protein in apo and ligand-bound form is in the compact state structure, (D) Density fluctuation of the simulation system as a function of the trajectory, with insignificant change, indicating that the environment of the solvent was well equilibrated, (E) The overall energy of the system is comparatively constant with some fluctuations which means that the system is in a thermodynamically stable state throughout the simulation, (F) SASA results indicating the solvent accessibility of the ligand bound complex is slightly smaller indicating that the complex undergoes changes in conformation on binding with the ligand, (G) The count of hydrogen bonds between the protein and the ligand in the process of simulation, representing the dynamic but stabilizing interactions as far as the stability of the complex, (H) PCA projection of the first two principal components (PC1 and PC2), which demonstrate that the largest part of the conformational states is concentrated in a rather narrow region, which indicates that the global dynamics are stable, (I) Residue-by-residue energy decomposition analysis (MM-PBSA) of the key residues involved in ligand binding due to favorable dynamics of the energy.

To evaluate the impact of ligand binding on MSLN structural dynamics, 100 ns molecular dynamics (MD) simulations were performed for both the apo MSLN protein and the MSLN-Withaferin A complex. The root mean square deviation (RMSD) analysis showed that both the apo and ligand-bound systems remained structurally stable throughout the simulation period, with fluctuations ranging between approximately 1.5–3.5 Å (Fig 11a). Overall RMSD profile showed that the protein did not show any significant structural destabilization on binding the ligand. Comparable flexibility patterns of both systems were revealed by root mean square fluctuation (RMSF) analysis where RMSF of the residues at the N- and C-terminal regions of backbone was increased, but RMSF of residues in the binding pocket region of the ligand bound system was relatively decreased indicating stabilization of the local region during the binding of the ligand (Fig 11b). The radius of gyration (Rg) was also found to be constant in both the systems during the simulation suggesting that the overall structure is compact, and there is no change in the compactness (Fig 11c). The fluctuations of the total energy and density profiles were small, and the systems were stabilized over the time scale of the simulation, indicating equilibrated systems (Figs 11d and 11e). The solvent accessible surface area (SASA) analysis also revealed that the protein with ligand brought about a small conformational change in the system due to ligand binding (Fig 11f). Hydrogen bond analysis showed dynamic and transient interaction with Withaferin A and MSLN all along the simulation (Fig 11g). The principal component analysis (PCA) showed that both systems explored a limited range of conformations with PC1 and PC2 explaining 34.64% and 19.76% of the total variance, respectively (Fig. 10h), indicating limited changes in large scale conformation. Finally, MM/PBSA analysis showed that the binding free energy contribution is favorable for MSLN-Withaferin A and key residue contributions are favorable for ligand stabilization (Fig 11i). Based on the comparative analysis of apo and ligand-bound systems, ligand binding appears to aid in the local stabilization of the MSLN structure but does not disrupt the global structure.

### 3.12. Prediction of drug-likeness, ADMET and toxicity of Withaferin A

Following docking and interaction analyses, the pharmacokinetic properties, drug-likeness characteristics, and toxicity profile of Withaferin A were evaluated to assess its suitability as a potential MSLN-targeting compound. SwissADME analysis revealed favorable pharmacokinetic characteristics for Withaferin A (Table 3). The compound had a topological polar surface area (TPSA) of 96.36 Å^2^, three rotatable bonds, and consensus LogP of 3.42 which is placing the compound in a moderate lipophilicity class. In addition, Withaferin A was predicted to have high gastrointestinal absorption and moderate water solubility, which indicate good oral bioavailability. Lipinski rule of five analysis revealed that the Withaferin A complies with most of the drug likeness criteria, but it showed 2 violations: Molecular weight >350 Da and XLOGP3 > 3.5 (Table 4). However, the compound exhibited desirable physicochemical/pharmacokinetic traits, which suggest the defects might not fully disqualify the potential drug development. ProTox-III predicted LD50 as 300 mg/kg, and categorized Withaferin A as toxicity class III compound suggesting moderate acute toxicity (Table 5). However, no hepatotoxic, mutagenic (Ame’s test) and carcinogenic toxicity endpoints were identified during the further toxicity endpoint prediction (Table 6). However, the compound was predicted to exhibit respiratory toxicity, cardiotoxicity, and immunotoxicity, highlighting potential safety concerns that should be considered during future preclinical development.

**Table 3:** ADMET results of Withaferin A.

Ligand	Filters					
	Physicochemical Properties		Lipophilicity	Pharmacokinetics	Water Solubility	Lead Likeness
	TPSA (Å^2^)	No. of Rotatable Bonds	Consensus Log p	GI Absorption		
Withaferin A	96.36	3	3.42	High	Moderately soluble	No; 2 violations: MW > 350, XLOGP3 > 3.5

**Table 4:** Lipinski’s rule of five compliance for selected Withaferin A compound.

Ligand Name	Properties				
	Mass, mg/mol	HBD	HBA	Log P	Molar refractivity
Withaferin A	470.60	6	2	2.75	127.49

**Table 5:** Predicted LD₅₀ values and toxicity class of the Withaferin A.

Compound	LD50 (mg/kg)	Toxicity class
Withaferin A	300	III

**Table 6:** In silico toxicity endpoint prediction of Withaferin A.

Compound	Toxicity endpoint	Prediction	Tool
Withaferin A	Hepatotoxicity	No	ProTox-III
	Mutagenicity (Ames)	No	ProTox-III
	Carcinogenicity	No	ProTox-III
	Respiratory toxicity	Yes	ProTox-III
	Cardiotoxicity	Yes	ProTox-III
	Immunotoxicity	Yes	ProTox-III

## 4. Discussion

Mesothelin (MSLN) is a well-known tumor associated with antigen restrictedly expressed in normal mesothelial tissue and markedly over-expressed in a variety of solid tumors. In the present study, the integrated transcriptomic analysis revealed that MSLN expression was associated with multiple cancer types including pancreatic adenocarcinoma (PAAD), mesothelioma (MESO), ovarian cancer (OV), lung adenocarcinoma (LUAD), etc. with elevated expression. The results support earlier studies that found MSLN to be clinically relevant as a biomarker and therapeutic target for solid tumors [4,5]. It incorporated epithelial and mesothelial malignancies with known cases of MSLN regulation and helped to interpret its biological significance in the selected subset of cancers by comparing its expression patterns between each type. Significant MSLN overexpression was also observed in PAAD by independent GEO validation, confirming reproducibility and the robustness of the transcriptomic results.

Diagnostic analysis revealed cancer dependency for MSLN. The strong discriminatory performance was observed in PAAD and STAD, while the diagnostic performance was moderate in CHOL and limited in LUAD and BRCA. This heterogeneity indicates that MSLN can be a lineage and context specific cancer marker rather than a universal marker. The same variation has been observed for other tumor-associated antigens and could result from different variations of tissue, microenvironment, and regulatory processes that contribute to the control of gene expression [30]. Stage-wise analysis also showed that the expression of MSLN was relatively unchanged with the progression of the tumors except in PAAD where expression was seen to be slightly up-regulated during later stages. This finding indicates that MSLN expressions are broadly related to the nature of the tumor and is not necessarily related to the continuous evolution of the disease. However, increased expression has been associated with advanced PAAD and could suggest a role in tumor aggressiveness and invasive behavior, as high expression of this gene has been linked to increased adhesion and invasion in pancreatic cancer models [9].

The prognostic value of MSLN also seemed to be cancer specific. Kaplan-Meier analysis revealed significant associations of high MSLN expression with poor overall survival in LUAD, MESO and OV but not in PAAD, CHOL, STAD and BRCA. Notably, Cox proportional hazards model revealed that MSLN was an independent prognostic factor in PAAD. This apparent discrepancy is almost certainly due to methodological differences in analytical approaches. Cox regression can account for clinical factors (covariates) and express the gene expression as a continuous variable, whereas Kaplan-Meier analysis will categorize patients into specific gene expression groups, potentially lowering the statistical power through dichotomization [31]. Hence the prognostic significance seen in PAAD might only be evident after taking clinical heterogeneity and confounding factors into consideration. The results highlight the strong dependency of MSLN-associated prognosis on the statistical methodology and tumor context. MSLN alteration proved to be relatively infrequent in different cancer types, and most alterations were missense mutations having a frequency of less or equal to 5%. Most effects were observed in the N-terminal part of the molecule, and no hot spot mutations were found. Modifications in the N-terminal domain of MSLN have been linked to changes in the extracellular function and protein interaction, and may, therefore, affect biological behavior even with low frequencies. However, the lack of any mutations at high prevalence or in a recurrent manner suggests that genome alteration is not likely the main mechanism of MSLN overexpression. However, regulation of expression is probably more heavily influenced by factors such as transcriptional activation, epigenetic regulation and signaling by the tumor microenvironment. However, analogous changes have been observed with other tumor antigens, which depend on transpositional regulation rather than direct mutation of their genes [32].

Functional enrichment analysis performed on the MSLN associated proteins identified from BioGRID provided significant enrichment in immune related processes and Tumor associated processes such as cytokine signaling, leukocyte activation and cell adhesion. Additional pathways that were identified through KEGG pathway analysis included cytokine-cytokine receptor interaction and JAK-STAT signaling pathways. These results suggest that MSLN may be involved in tumor progression, as a regulator of the immune system or as a mediator of intercellular communication in the tumor microenvironment and not as a classical oncogenic driver. Similarly, previous studies have suggested that MSLN may interact with immune and stromal cells that regulate tumor progression [33]. Enrichment in extracellular and membrane associated components further confirmed its known functions as cell surface glycoprotein in cellular signaling and tumor microenvironmental communication.

Phytochemical screening targeting MSLN was carried out due to its constant overexpression and membrane localization. The result of molecular docking revealed that Withaferin A scored the most favorable binding affinity within all screened phytochemicals as well as the reference ligand, Doxorubicin, by both AutoDock Vina and Glide scoring systems. Interestingly, both the hydrophilic and lipophilic compounds were able to display good docking scores, indicating that the ability to dock with the predicted MSLN binding cavity is not solely dependent on the global physicochemical character of the compound, but also the complementary steric and electrostatic interactions within the MSLN binding cavity. The hydrogen bonding and hydrophobic contacts with residues lining the predicted cavity were determined by interaction analysis, which confirmed stable interactions.

Therefore, docking results must be treated with caution as the hypothesis generated from the docking results should be further validated by experiments. The molecular dynamics simulations also revealed the stable conformational behavior of both apo and ligand-bound systems throughout the 100 ns trajectory, further supporting the interaction between MSLN and Withaferin A. The results of the RMSD, RMSF, radius of gyration, and energy analysis all demonstrated that the structural integrity and local stabilization was retained upon ligand binding. The flexibility of the residues of the binding site, accompanied by restricted conformational sampling uncovered by PCA, supported the stable accommodation of the ligand. A close hydrogen bonding and favorable MMPBSA binding energies further suggested the nature of the interaction mode predicted. The consistency of the docking, molecular dynamics and free-energy analyses provides confidence in the proposed binding model, but such computational analysis is still predictive and replaces neither biochemical nor cellular confirmation [34].

An in-silico drug-likeness analysis was conducted to gain insights into the translational potential of Withaferin A, and SwissADME predictions indicated good gastrointestinal absorption, moderate lipophilicity, and acceptable solubility suggesting potential oral bioavailability. While some partial Lipinski’s violations were noted, it is common for natural products with anticancer properties to exceed the Lipinski’s properties and it does not preclude therapeutic importance. However, moderate acute toxicity, as well as potential cardiotoxicity, respiratory toxicity, and immunotoxicity were predicted by ProTox-III analysis. These findings pose clinically important issues and indicate that the direct use of Withaferin A as therapy might be limited by concerns of safety. These liabilities do not exclude therapeutic potential but still underline the necessity of structural optimization of these agents, as well as their derivatization to enhance pharmacological selectivity and off-target toxicity. Such optimization approaches have been successful in the case of steroidal lactones and natural-product scaffolds related to this class [35]. Therefore, further research is required to perform in vitro cytotoxicity and safety testing to determine if improved safety profiles can be accomplished with optimized derivatives that maintain affinity for MSLN.

There are several limitations that should be noted. First, all the transcriptomic analyses in the present study are in silico and public and need to be validated in cellular and animal models. Secondly, the actual binding pocket of MSLN and its ligand interactions have yet to be validated. Third, molecular dynamics had been simulated to 100 ns and may not fully represent the conformational behavior on longer time scales. Lastly, ADMET and toxicity predictions are only estimates and do not necessarily reflect the biological complexity. Altogether, this study indicates that MSLN is a biologically relevant but context-dependent biomarker with diagnostic and therapeutic significance especially in PAAD and mesothelioma. An integrated transcriptomic, functional, and structural approach identified Withaferin A as a potentially interacting compound that is supported by docking, molecular dynamics, and free-energy calculations of the compound with MSLN. But the predicted toxicity and lack of experimental validation suggest that these results are exploratory and should be followed up biologically and pharmacologically prior to translation for therapy.

## 5. Conclusion

MSLN is a cancer-associated biomarker that is consistently over-expressed in several types of cancers, especially pancreatic adenocarcinoma, mesothelioma and ovarian cancer. In this study, multi-omics analyses have confirmed that MSLN has cancer-type specific diagnostic potential, effective in pancreatic and stomach cancers and less effective in other cancers. Survival and genomic studies suggest a heterogeneous prognostic significance and that the dysregulation of MSLN is primarily occurring at the transcriptional or epigenetic level and not by recurrent mutational events. Functional enrichment analysis also confirmed that MSLN is involved with immune regulation, tumor-microenvironment signaling pathways, but do not directly imply an oncogenic mutation. In the drug sensitivity analysis, moderate correlations with the expression of MSLN and sensitivity to selected drugs are shown, potential and require further validation. Structure-based screening identified Withaferin A as a promising MSLN-binding compound, supported by docking, molecular dynamics, and binding free energy analyses. But based on predicted toxicity and pharmacokinetic limitations, direct translation into the clinic is not possible unless further optimization is performed. Overall, the results from this study suggest that MSLN is a potentially biologically relevant diagnostic and context specific marker with potential therapeutic utility in certain cancers, especially pancreatic adenocarcinoma. The results suggest further experimental validation and rational design of drugs targeting MSLN for future applications in translation.

## Supporting information

S1 Table Complete list of BioGRID-derived MSLN interacting proteins. (DOCX)

S2 Table MSLN expressions in the tumor and normal human samples of the selected TCGA datasets. (CSV)

S3 Table MSLN expressions in the tumor and normal human samples of the GSE15471 dataset. (DOCX)

S4 Table MSLN expressions in the tumor and normal human samples of the GSE62452 dataset. (DOCX)

S5 Table Multivariate Cox values of the MSLN in the selected cancer types. (DOCX)

S6 Table Biological processes are associated with MSLN-interacted genes. (DOCX)

S7 Table Molecular functions are associated with MSLN-interacted proteins. (DOCX)

S8 Table Cellular components are associated with MSLN-interacted proteins. (DOCX)

S9 Table Reactome pathways are associated with the MSLN-interacted genes. (DOCX)

S10 Table Wiki pathways are associated with MSLN-interacted genes. (DOCX)

S11 Table KEGG pathways are associated with the MSLN-interacted proteins. (DOCX)

S12 Table GDSC drug-gene correlation analysis for MSLN. (DOCX)

S13 Table CTRP drug–gene correlation analysis for MSLN. (DOCX)

S14 Table Binding affinities of all complexes and phytochemicals class. (DOCX)

S1 File R script used for pan-cancer analysis of MSLN expression and associated clinicopathological profiling across TCGA datasets. (TXT)

S2 File R script used for validation analyses of MSLN expression and diagnostic performance using GEO datasets. (TXT)

S3 File R script used for mutation and genomic alteration analyses of MSLN across cancer types. (TXT)
