# Supplementary material for: Pan-cancer profiles of mesothelin (MSLN) unveil diagnostic potential and therapeutic targetability of pancreatic adenocarcinoma
Source: PLoS One. 2026 Aug 20;21(8):e0354334. doi: 10.1371/journal.pone.0354334 (PMC13492821; doi:10.1371/journal.pone.0354334)
Supplement: Supplementary file 1 [file pone.0354334.s001.docx]

**Table S1.** Complete list of BioGRID-derived MSLN interacting proteins.

| **Sl. No.** | **Interacting Protein** | | |
| --- | --- | --- | --- |
| 1 | DDX31 | 45 | MMGT1 |
| 2 | ACPT | 46 | MPV17 |
| 3 | ANLN | 47 | MRPL1 |
| 4 | APEX1 | 48 | MRPL16 |
| 5 | APPBP2 | 49 | MRPL28 |
| 6 | ASIC5 | 50 | NAALADL1 |
| 7 | ATP6V1A | 51 | NCR3 |
| 8 | BMP2 | 52 | NDUFB5 |
| 9 | BPIFB6 | 53 | NDUFB9 |
| 10 | BSPRY | 54 | NODAL |
| 11 | BTF3 | 55 | PCGF2 |
| 12 | C19ORF80 | 56 | POU2AF1 |
| 13 | CA3 | 57 | PPY |
| 14 | CBLN4 | 58 | PRG2 |
| 15 | CCL3 | 59 | PRSS16 |
| 16 | DEFB109 | 60 | RAG2 |
| 17 | DEFB135 | 61 | RALA |
| 18 | DNASE1L1 | 62 | RING1 |
| 19 | ECEL1 | 63 | RNF157 |
| 20 | EMC1 | 64 | RNMTL1 |
| 21 | EMC2 | 65 | RPL29 |
| 22 | FAM150B | 66 | RSG1 |
| 23 | FKBP14 | 67 | SDF2L1 |
| 24 | FUT3 | 68 | SFTPC |
| 25 | GGH | 69 | SLC48A1 |
| 26 | GKN2 | 70 | TGOLN2 |
| 27 | GOLGA8A | 71 | TMTC2 |
| 28 | GXYLT1 | 72 | TRHDE |
| 29 | HDAC6 | 73 | TRIM25 |
| 30 | HLA-G | 74 | UGT2B28 |
| 31 | IFNA16 | 75 | ULBP1 |
| 32 | IFNK | 76 | WFDC1 |
| 33 | IL5RA | 77 | ZBTB43 |
| 34 | KLK9 | 78 | ZC2HC1B |
| 35 | KRT35 | 79 | ZDHHC23 |
| 36 | LAMTOR3 | 80 | ZPBP2 |
| 37 | LARP1B | 81 | MSLN |
| 38 | LY6G5B |  |  |
| 39 | LY6G5C |  |  |
| 40 | LY86 |  |  |
| 41 | LYPD3 |  |  |
| 42 | LYPD4 |  |  |
| 43 | MAMDC4 |  |  |
| 44 | MFNG |  |  |
