# Supplementary material for: Pan-cancer profiles of mesothelin (MSLN) unveil diagnostic potential and therapeutic targetability of pancreatic adenocarcinoma
Source: PLoS One. 2026 Aug 20;21(8):e0354334. doi: 10.1371/journal.pone.0354334 (PMC13492821; doi:10.1371/journal.pone.0354334)
Supplement: Supplementary file 2 [file pone.0354334.s003.docx]

**Table S3.** MSLN expressions in the tumor and normal human samples of the GSE15471 dataset.

| **Sample** | **Expression** | **Type** |
| --- | --- | --- |
| GSM388076 | 4.342678 | Normal |
| GSM388077 | 4.248201 | Normal |
| GSM388078 | 4.703837 | Normal |
| GSM388079 | 4.719449 | Normal |
| GSM388080 | 5.175197 | Normal |
| GSM388081 | 5.078351 | Normal |
| GSM388082 | 5.35835 | Normal |
| GSM388083 | 4.128759 | Normal |
| GSM388084 | 4.794804 | Normal |
| GSM388085 | 5.647518 | Normal |
| GSM388086 | 4.478487 | Normal |
| GSM388087 | 4.79631 | Normal |
| GSM388088 | 4.439735 | Normal |
| GSM388089 | 4.314469 | Normal |
| GSM388090 | 5.196486 | Normal |
| GSM388091 | 4.948699 | Normal |
| GSM388092 | 5.005333 | Normal |
| GSM388093 | 5.219367 | Normal |
| GSM388094 | 4.323297 | Normal |
| GSM388095 | 5.191634 | Normal |
| GSM388096 | 5.287276 | Normal |
| GSM388097 | 5.237651 | Normal |
| GSM388098 | 4.425053 | Normal |
| GSM388099 | 4.649367 | Normal |
| GSM388100 | 4.641021 | Normal |
| GSM388101 | 5.557309 | Normal |
| GSM388102 | 4.730154 | Normal |
| GSM388103 | 5.178012 | Normal |
| GSM388104 | 4.482276 | Normal |
| GSM388105 | 4.488387 | Normal |
| GSM388106 | 5.191029 | Normal |
| GSM388107 | 5.1033 | Normal |
| GSM388108 | 5.149232 | Normal |
| GSM388109 | 5.297263 | Normal |
| GSM388110 | 4.984221 | Normal |
| GSM388111 | 6.094938 | Normal |
| GSM388112 | 5.269372 | Normal |
| GSM388113 | 5.200573 | Normal |
| GSM388114 | 4.539168 | Normal |
| GSM388115 | 5.157563 | Tumor |
| GSM388116 | 5.82057 | Tumor |
| GSM388117 | 6.41613 | Tumor |
| GSM388118 | 5.955043 | Tumor |
| GSM388119 | 7.55045 | Tumor |
| GSM388120 | 7.966883 | Tumor |
| GSM388121 | 10.57184 | Tumor |
| GSM388122 | 5.491712 | Tumor |
| GSM388123 | 4.884567 | Tumor |
| GSM388124 | 6.120678 | Tumor |
| GSM388125 | 6.145082 | Tumor |
| GSM388126 | 6.899656 | Tumor |
| GSM388127 | 4.320401 | Tumor |
| GSM388128 | 4.720087 | Tumor |
| GSM388129 | 7.096271 | Tumor |
| GSM388130 | 4.190401 | Tumor |
| GSM388131 | 5.184882 | Tumor |
| GSM388132 | 5.181117 | Tumor |
| GSM388133 | 4.696807 | Tumor |
| GSM388134 | 4.555071 | Tumor |
| GSM388135 | 7.733136 | Tumor |
| GSM388136 | 5.717948 | Tumor |
| GSM388137 | 4.786032 | Tumor |
| GSM388138 | 7.165909 | Tumor |
| GSM388139 | 8.25913 | Tumor |
| GSM388140 | 6.972106 | Tumor |
| GSM388141 | 5.695811 | Tumor |
| GSM388142 | 8.953556 | Tumor |
| GSM388143 | 8.821236 | Tumor |
| GSM388144 | 8.279155 | Tumor |
| GSM388145 | 5.624144 | Tumor |
| GSM388146 | 8.324575 | Tumor |
| GSM388147 | 5.299839 | Tumor |
| GSM388148 | 7.832311 | Tumor |
| GSM388149 | 9.012977 | Tumor |
| GSM388150 | 7.192714 | Tumor |
| GSM388151 | 6.737784 | Tumor |
| GSM388152 | 6.119858 | Tumor |
| GSM388153 | 6.754584 | Tumor |
