# Supplementary material for: Pan-cancer profiles of mesothelin (MSLN) unveil diagnostic potential and therapeutic targetability of pancreatic adenocarcinoma
Source: PLoS One. 2026 Aug 20;21(8):e0354334. doi: 10.1371/journal.pone.0354334 (PMC13492821; doi:10.1371/journal.pone.0354334)
Supplement: Supplementary file 3 [file pone.0354334.s004.docx]

**Table S4.** MSLN expressions in the tumor and normal human samples of the GSE62452 dataset.

| **Sample** | **Expression** | **Type** |
| --- | --- | --- |
| GSM1527106 | 4.60352 | Normal |
| GSM1527108 | 4.07399 | Normal |
| GSM1527110 | 4.06695 | Normal |
| GSM1527112 | 3.653 | Normal |
| GSM1527114 | 3.41001 | Normal |
| GSM1527116 | 4.18622 | Normal |
| GSM1527118 | 3.74219 | Normal |
| GSM1527120 | 4.26604 | Normal |
| GSM1527122 | 3.89515 | Normal |
| GSM1527124 | 4.00317 | Normal |
| GSM1527126 | 4.163 | Normal |
| GSM1527128 | 3.78131 | Normal |
| GSM1527130 | 3.97883 | Normal |
| GSM1527132 | 3.27391 | Normal |
| GSM1527134 | 4.20101 | Normal |
| GSM1527136 | 4.18376 | Normal |
| GSM1527138 | 3.84774 | Normal |
| GSM1527140 | 3.77327 | Normal |
| GSM1527142 | 3.74567 | Normal |
| GSM1527144 | 3.77036 | Normal |
| GSM1527146 | 3.90628 | Normal |
| GSM1527148 | 3.85142 | Normal |
| GSM1527150 | 4.09806 | Normal |
| GSM1527152 | 3.92321 | Normal |
| GSM1527154 | 3.93591 | Normal |
| GSM1527156 | 4.98398 | Normal |
| GSM1527158 | 3.5464 | Normal |
| GSM1527160 | 4.50428 | Normal |
| GSM1527162 | 4.06985 | Normal |
| GSM1527164 | 3.86191 | Normal |
| GSM1527166 | 4.22643 | Normal |
| GSM1527168 | 3.59091 | Normal |
| GSM1527170 | 3.59946 | Normal |
| GSM1527172 | 3.69152 | Normal |
| GSM1527174 | 4.4481 | Normal |
| GSM1527176 | 3.88362 | Normal |
| GSM1527178 | 3.72887 | Normal |
| GSM1527180 | 3.72704 | Normal |
| GSM1527182 | 4.26805 | Normal |
| GSM1527184 | 3.93299 | Normal |
| GSM1527186 | 3.4872 | Normal |
| GSM1527188 | 3.92581 | Normal |
| GSM1527190 | 3.79181 | Normal |
| GSM1527192 | 3.61157 | Normal |
| GSM1527194 | 3.46687 | Normal |
| GSM1527195 | 4.28348 | Normal |
| GSM1527197 | 3.55949 | Normal |
| GSM1527201 | 4.86295 | Normal |
| GSM1527203 | 3.89368 | Normal |
| GSM1527206 | 3.85688 | Normal |
| GSM1527208 | 4.77244 | Normal |
| GSM1527211 | 5.791 | Normal |
| GSM1527214 | 4.04714 | Normal |
| GSM1527217 | 4.03286 | Normal |
| GSM1527221 | 4.22298 | Normal |
| GSM1527222 | 4.65909 | Normal |
| GSM1527224 | 3.6204 | Normal |
| GSM1527226 | 4.18905 | Normal |
| GSM1527229 | 3.77114 | Normal |
| GSM1527231 | 3.80707 | Normal |
| GSM1527233 | 4.11299 | Normal |
| GSM1527105 | 5.53003 | Tumor |
| GSM1527107 | 5.00188 | Tumor |
| GSM1527109 | 3.86179 | Tumor |
| GSM1527111 | 4.01598 | Tumor |
| GSM1527113 | 5.62604 | Tumor |
| GSM1527115 | 5.43734 | Tumor |
| GSM1527117 | 5.13229 | Tumor |
| GSM1527119 | 5.11164 | Tumor |
| GSM1527121 | 4.98768 | Tumor |
| GSM1527123 | 4.98756 | Tumor |
| GSM1527125 | 4.70975 | Tumor |
| GSM1527127 | 4.30229 | Tumor |
| GSM1527129 | 4.93206 | Tumor |
| GSM1527131 | 3.79571 | Tumor |
| GSM1527133 | 3.90627 | Tumor |
| GSM1527135 | 4.55198 | Tumor |
| GSM1527137 | 4.60771 | Tumor |
| GSM1527139 | 3.74413 | Tumor |
| GSM1527141 | 4.51071 | Tumor |
| GSM1527143 | 6.23037 | Tumor |
| GSM1527145 | 3.78565 | Tumor |
| GSM1527147 | 3.62827 | Tumor |
| GSM1527149 | 4.9275 | Tumor |
| GSM1527151 | 5.1155 | Tumor |
| GSM1527153 | 5.94265 | Tumor |
| GSM1527155 | 5.62464 | Tumor |
| GSM1527157 | 5.67039 | Tumor |
| GSM1527159 | 6.68746 | Tumor |
| GSM1527161 | 4.64497 | Tumor |
| GSM1527163 | 5.52377 | Tumor |
| GSM1527165 | 5.51402 | Tumor |
| GSM1527167 | 4.20921 | Tumor |
| GSM1527169 | 4.82721 | Tumor |
| GSM1527171 | 4.78065 | Tumor |
| GSM1527173 | 5.19228 | Tumor |
| GSM1527175 | 3.98383 | Tumor |
| GSM1527177 | 5.27515 | Tumor |
| GSM1527179 | 4.38731 | Tumor |
| GSM1527181 | 5.30289 | Tumor |
| GSM1527183 | 5.13289 | Tumor |
| GSM1527185 | 4.27131 | Tumor |
| GSM1527187 | 6.17713 | Tumor |
| GSM1527189 | 4.52951 | Tumor |
| GSM1527191 | 3.98938 | Tumor |
| GSM1527193 | 4.91465 | Tumor |
| GSM1527196 | 5.54634 | Tumor |
| GSM1527198 | 5.79472 | Tumor |
| GSM1527199 | 4.18331 | Tumor |
| GSM1527200 | 3.37638 | Tumor |
| GSM1527202 | 4.9504 | Tumor |
| GSM1527204 | 5.47212 | Tumor |
| GSM1527205 | 3.70244 | Tumor |
| GSM1527207 | 4.61311 | Tumor |
| GSM1527209 | 5.06831 | Tumor |
| GSM1527210 | 6.38702 | Tumor |
| GSM1527212 | 4.24146 | Tumor |
| GSM1527213 | 6.49379 | Tumor |
| GSM1527215 | 3.44857 | Tumor |
| GSM1527216 | 3.93893 | Tumor |
| GSM1527218 | 4.41758 | Tumor |
| GSM1527219 | 4.44172 | Tumor |
| GSM1527220 | 6.34643 | Tumor |
| GSM1527223 | 4.78581 | Tumor |
| GSM1527225 | 6.21021 | Tumor |
| GSM1527227 | 4.36914 | Tumor |
| GSM1527228 | 3.81022 | Tumor |
| GSM1527230 | 3.70483 | Tumor |
| GSM1527232 | 3.77663 | Tumor |
| GSM1527234 | 4.53848 | Tumor |
