# Supplementary material for: Pan-cancer profiles of mesothelin (MSLN) unveil diagnostic potential and therapeutic targetability of pancreatic adenocarcinoma
Source: PLoS One. 2026 Aug 20;21(8):e0354334. doi: 10.1371/journal.pone.0354334 (PMC13492821; doi:10.1371/journal.pone.0354334)
Supplement: Supplementary file 4 [file pone.0354334.s005.docx]

**Table S5.** Multivariate Cox values of the MSLN in the selected cancer types.

| Cancer | HR | Lower95 | Upper95 | P_value |
| --- | --- | --- | --- | --- |
| PAAD | 1.147964 | 1.059393 | 1.24394 | 0.000756 |
| CHOL | 1.00601 | 0.913954 | 1.107337 | 0.902606 |
| STAD | 0.97228 | 0.928477 | 1.018149 | 0.231996 |
| LUAD | 1.030157 | 0.986779 | 1.075441 | 0.175858 |
| OV | 1.054184 | 0.967959 | 1.14809 | 0.225516 |
| MESO | 0.831834 | 0.755369 | 0.91604 | 0.000182 |
| BRCA | 0.998617 | 0.953984 | 1.045338 | 0.952685 |
