# Supplementary material for: Pan-cancer profiles of mesothelin (MSLN) unveil diagnostic potential and therapeutic targetability of pancreatic adenocarcinoma
Source: PLoS One. 2026 Aug 20;21(8):e0354334. doi: 10.1371/journal.pone.0354334 (PMC13492821; doi:10.1371/journal.pone.0354334)
Supplement: Supplementary file 5 [file pone.0354334.s006.docx]

**Table S6.** Biological processes are associated with MSLN-interacted genes.

| **Index** | **Name** | **P-value** | **Adjusted p-value** | **Odds Ratio** | **Combined score** |
| --- | --- | --- | --- | --- | --- |
| 1 | Protein Insertion into ER Membrane by Stop-Transfer Membrane-Anchor Sequence (GO:0045050) | 0.00001031 | 0.006663 | 95.73 | 1099.12 |
| 2 | Tail-anchored Membrane Protein Insertion into ER Membrane (GO:0071816) | 0.00004177 | 0.01162 | 54.68 | 551.40 |
| 3 | Natural Killer Cell Activation (GO:0030101) | 0.00005398 | 0.01162 | 21.96 | 215.84 |
| 4 | Protein Insertion into ER Membrane (GO:0045048) | 0.0002651 | 0.04281 | 27.32 | 225.02 |
| 5 | Positive Regulation of Leukocyte Mediated Cytotoxicity (GO:0001912) | 0.001368 | 0.1062 | 14.98 | 98.81 |
| 6 | Antigen Processing and Presentation of Endogenous Peptide Antigen via MHC Class I via ER Pathway (GO:0002484) | 0.001428 | 0.1062 | 42.00 | 275.14 |
| 7 | Ag Proc and Presentation of Endogenous Pep Ag via MHC Class I via ER Pway, TAP-ind (GO:0002486) | 0.001428 | 0.1062 | 42.00 | 275.14 |
| 8 | Antigen Processing and Presentation of Endogenous Peptide Antigen via MHC Class Ib (GO:0002476) | 0.001428 | 0.1062 | 42.00 | 275.14 |
| 9 | Antigen Processing and Presentation of Peptide Antigen via MHC Class Ib (GO:0002428) | 0.001644 | 0.1062 | 38.77 | 248.51 |
| 10 | Mature B Cell Differentiation Involved in Immune Response (GO:0002313) | 0.001644 | 0.1062 | 38.77 | 248.51 |
| 11 | Cellular Response to Virus (GO:0098586) | 0.002038 | 0.1140 | 12.95 | 80.22 |
| 12 | Peptide Catabolic Process (GO:0043171) | 0.002118 | 0.1140 | 33.59 | 206.84 |
| 13 | Antigen Processing and Presentation of Endogenous Peptide Antigen (GO:0002483) | 0.003551 | 0.1704 | 25.19 | 142.08 |
| 14 | Natural Killer Cell Activation Involved in Immune Response (GO:0002323) | 0.003879 | 0.1704 | 23.99 | 133.19 |
| 15 | Lymphocyte Activation Involved in Immune Response (GO:0002285) | 0.004220 | 0.1704 | 22.90 | 125.20 |
| 16 | DNA Catabolic Process (GO:0006308) | 0.004220 | 0.1704 | 22.90 | 125.20 |
| 17 | Response to dsRNA (GO:0043331) | 0.004575 | 0.1739 | 21.90 | 117.98 |
| 18 | Protein O-linked Glycosylation (GO:0006493) | 0.005484 | 0.1774 | 8.97 | 46.72 |
| 19 | Positive Regulation of Natural Killer Cell Mediated Immunity (GO:0002717) | 0.006127 | 0.1774 | 18.65 | 95.03 |
| 20 | Regulation of Androgen Receptor Signaling Pathway (GO:0060765) | 0.006981 | 0.1774 | 17.36 | 86.20 |
| 21 | Response to Exogenous dsRNA (GO:0043330) | 0.007885 | 0.1774 | 16.24 | 78.65 |
| 22 | B Cell Activation Involved in Immune Response (GO:0002312) | 0.007885 | 0.1774 | 16.24 | 78.65 |
| 23 | Mitochondrial Translation (GO:0032543) | 0.008022 | 0.1774 | 7.78 | 37.54 |
| 24 | Positive Regulation of T Cell Mediated Cytotoxicity (GO:0001916) | 0.008356 | 0.1774 | 15.73 | 75.28 |
| 25 | B Cell Activation (GO:0042113) | 0.008464 | 0.1774 | 7.62 | 36.38 |
| 26 | Mitochondrial Gene Expression (GO:0140053) | 0.008690 | 0.1774 | 7.55 | 35.81 |
| 27 | T Cell Activation Involved in Immune Response (GO:0002286) | 0.008840 | 0.1774 | 15.26 | 72.14 |
| 28 | Regulation of T Cell Mediated Cytotoxicity (GO:0001914) | 0.009843 | 0.1774 | 14.38 | 66.46 |
| 29 | Cellular Response to Acid Chemical (GO:0071229) | 0.009843 | 0.1774 | 14.38 | 66.46 |
| 30 | Response to Amino Acid (GO:0043200) | 0.01089 | 0.1774 | 13.60 | 61.48 |
| 31 | Regulation of Natural Killer Cell Mediated Cytotoxicity (GO:0042269) | 0.01089 | 0.1774 | 13.60 | 61.48 |
| 32 | Positive Regulation of SMAD Protein Signal Transduction (GO:0060391) | 0.01144 | 0.1774 | 13.25 | 59.22 |
| 33 | Negative Regulation of Fat Cell Differentiation (GO:0045599) | 0.01199 | 0.1774 | 12.90 | 57.08 |
| 34 | Mitochondrial Electron Transport, NADH to Ubiquinone (GO:0006120) | 0.01256 | 0.1774 | 12.58 | 55.07 |
| 35 | Type I Interferon-Mediated Signaling Pathway (GO:0060337) | 0.01373 | 0.1774 | 11.98 | 51.38 |
| 36 | Cellular Response to Amino Acid Stimulus (GO:0071230) | 0.01557 | 0.1774 | 11.18 | 46.54 |
| 37 | Positive Regulation of Response to Biotic Stimulus (GO:0002833) | 0.01557 | 0.1774 | 11.18 | 46.54 |
| 38 | Regulation of Cell-Cell Adhesion (GO:0022407) | 0.01620 | 0.1774 | 10.94 | 45.09 |
| 39 | Immune Response-Regulating Cell Surface Receptor Signaling Pathway (GO:0002768) | 0.01620 | 0.1774 | 10.94 | 45.09 |
| 40 | Positive Regulation of T Cell Mediated Immunity (GO:0002711) | 0.01750 | 0.1774 | 10.48 | 42.40 |
| 41 | Positive Regulation of Cell-Cell Adhesion (GO:0022409) | 0.01817 | 0.1774 | 10.27 | 41.15 |
| 42 | Translation (GO:0006412) | 0.01898 | 0.1774 | 4.14 | 16.40 |
| 43 | Negative Regulation of G0 to G1 Transition (GO:0070317) | 0.02009 | 0.1774 | 62.23 | 243.19 |
| 44 | Positive Regulation of T Cell Tolerance Induction (GO:0002666) | 0.02009 | 0.1774 | 62.23 | 243.19 |
| 45 | Aggresome Assembly (GO:0070842) | 0.02009 | 0.1774 | 62.23 | 243.19 |
| 46 | Forebrain Regionalization (GO:0021871) | 0.02009 | 0.1774 | 62.23 | 243.19 |
| 47 | Semi-lunar Valve Development (GO:1905314) | 0.02009 | 0.1774 | 62.23 | 243.19 |
| 48 | Positive Regulation of Natural Killer Cell Chemotaxis (GO:2000503) | 0.02009 | 0.1774 | 62.23 | 243.19 |
| 49 | Regulation of Hydrogen Peroxide Metabolic Process (GO:0010310) | 0.02009 | 0.1774 | 62.23 | 243.19 |
| 50 | Neuroendocrine Cell Differentiation (GO:0061101) | 0.02009 | 0.1774 | 62.23 | 243.19 |
| 51 | Type 2 Mitophagy (GO:0061734) | 0.02009 | 0.1774 | 62.23 | 243.19 |
| 52 | Pre-B Cell Differentiation (GO:0002329) | 0.02009 | 0.1774 | 62.23 | 243.19 |
| 53 | Regulation of Protein Localization to Endoplasmic Reticulum (GO:1905550) | 0.02009 | 0.1774 | 62.23 | 243.19 |
| 54 | Eosinophil Activation Involved in Immune Response (GO:0002278) | 0.02009 | 0.1774 | 62.23 | 243.19 |
| 55 | Eosinophil Degranulation (GO:0043308) | 0.02009 | 0.1774 | 62.23 | 243.19 |
| 56 | Eosinophil Mediated Immunity (GO:0002447) | 0.02009 | 0.1774 | 62.23 | 243.19 |
| 57 | Nucleic Acid Catabolic Process (GO:0141188) | 0.02094 | 0.1774 | 9.49 | 36.69 |
| 58 | Negative Regulation of Gene Expression, Epigenetic (GO:0045814) | 0.02149 | 0.1774 | 5.32 | 20.43 |
| 59 | Interferon-mediated Signaling Pathway (GO:0140888) | 0.02313 | 0.1774 | 8.98 | 33.82 |
| 60 | Negative Regulation of Cardiac Muscle Cell Differentiation (GO:2000726) | 0.02406 | 0.1774 | 49.78 | 185.56 |
| 61 | Glial Cell Migration (GO:0008347) | 0.02406 | 0.1774 | 49.78 | 185.56 |
| 62 | Negative Regulation of Dendritic Cell Differentiation (GO:2001199) | 0.02406 | 0.1774 | 49.78 | 185.56 |
| 63 | Heme Transport (GO:0015886) | 0.02406 | 0.1774 | 49.78 | 185.56 |
| 64 | Negative Regulation of Insulin-Like Growth Factor Receptor Signaling Pathway (GO:0043569) | 0.02406 | 0.1774 | 49.78 | 185.56 |
| 65 | Negative Regulation of Leukocyte Differentiation (GO:1902106) | 0.02406 | 0.1774 | 49.78 | 185.56 |
| 66 | Septin Cytoskeleton Organization (GO:0032185) | 0.02406 | 0.1774 | 49.78 | 185.56 |
| 67 | Septin Ring Organization (GO:0031106) | 0.02406 | 0.1774 | 49.78 | 185.56 |
| 68 | Positive Regulation of Microglial Cell Activation (GO:1903980) | 0.02406 | 0.1774 | 49.78 | 185.56 |
| 69 | Positive Regulation of Microglial Cell Migration (GO:1904141) | 0.02406 | 0.1774 | 49.78 | 185.56 |
| 70 | Positive Regulation of Natural Killer Cell Cytokine Production (GO:0002729) | 0.02406 | 0.1774 | 49.78 | 185.56 |
| 71 | Iron Coordination Entity Transport (GO:1901678) | 0.02406 | 0.1774 | 49.78 | 185.56 |
| 72 | Positive Regulation of Neuroepithelial Cell Differentiation (GO:1902913) | 0.02406 | 0.1774 | 49.78 | 185.56 |
| 73 | Positive Regulation of Odontoblast Differentiation (GO:1901331) | 0.02406 | 0.1774 | 49.78 | 185.56 |
| 74 | Positive Regulation of Osteoblast Proliferation (GO:0033690) | 0.02406 | 0.1774 | 49.78 | 185.56 |
| 75 | Positive Regulation of Peroxisome Proliferator Activated Receptor Signaling Pathway (GO:0035360) | 0.02406 | 0.1774 | 49.78 | 185.56 |
| 76 | Negative Regulation of Trophoblast Cell Migration (GO:1901164) | 0.02406 | 0.1774 | 49.78 | 185.56 |
| 77 | Regulation of Natural Killer Cell Cytokine Production (GO:0002727) | 0.02406 | 0.1774 | 49.78 | 185.56 |
| 78 | Protection From Natural Killer Cell Mediated Cytotoxicity (GO:0042270) | 0.02406 | 0.1774 | 49.78 | 185.56 |
| 79 | Protein O-linked Glycosylation via Glucose (GO:0180059) | 0.02406 | 0.1774 | 49.78 | 185.56 |
| 80 | Podocyte Cell Migration (GO:0090521) | 0.02406 | 0.1774 | 49.78 | 185.56 |
| 81 | Positive Regulation of TORC1 Signaling (GO:1904263) | 0.02464 | 0.1774 | 8.67 | 32.11 |
| 82 | Regulation of Bone Mineralization (GO:0030500) | 0.02541 | 0.1774 | 8.52 | 31.30 |
| 83 | Regulation of SMAD Protein Signal Transduction (GO:0060390) | 0.02619 | 0.1774 | 8.38 | 30.52 |
| 84 | Positive Regulation of ERK1 and ERK2 Cascade (GO:0070374) | 0.02636 | 0.1774 | 4.90 | 17.83 |
| 85 | Proton Motive Force-Driven Mitochondrial ATP Synthesis (GO:0042776) | 0.02698 | 0.1774 | 8.24 | 29.77 |
| 86 | RIG-I Signaling Pathway (GO:0039529) | 0.02801 | 0.1774 | 41.49 | 148.32 |
| 87 | Positive Regulation of Animal Organ Morphogenesis (GO:0110110) | 0.02801 | 0.1774 | 41.49 | 148.32 |
| 88 | Negative Regulation of Cardiocyte Differentiation (GO:1905208) | 0.02801 | 0.1774 | 41.49 | 148.32 |
| 89 | Antigen Processing and Presentation of Exogenous Peptide Antigen via MHC Class Ib (GO:0002477) | 0.02801 | 0.1774 | 41.49 | 148.32 |
| 90 | Regulation of T Cell Tolerance Induction (GO:0002664) | 0.02801 | 0.1774 | 41.49 | 148.32 |
| 91 | Regulation of Aldosterone Biosynthetic Process (GO:0032347) | 0.02801 | 0.1774 | 41.49 | 148.32 |
| 92 | Response to Increased Oxygen Levels (GO:0036296) | 0.02801 | 0.1774 | 41.49 | 148.32 |
| 93 | Granzyme-mediated Apoptotic Signaling Pathway (GO:0008626) | 0.02801 | 0.1774 | 41.49 | 148.32 |
| 94 | Inclusion Body Assembly (GO:0070841) | 0.02801 | 0.1774 | 41.49 | 148.32 |
| 95 | Regulation of Cortisol Biosynthetic Process (GO:2000064) | 0.02801 | 0.1774 | 41.49 | 148.32 |
| 96 | Negative Regulation of Steroid Metabolic Process (GO:0045939) | 0.02801 | 0.1774 | 41.49 | 148.32 |
| 97 | Cilium Disassembly (GO:0061523) | 0.02801 | 0.1774 | 41.49 | 148.32 |
| 98 | Regulation of Lymphocyte Mediated Immunity (GO:0002706) | 0.02801 | 0.1774 | 41.49 | 148.32 |
| 99 | Marginal Zone B Cell Differentiation (GO:0002315) | 0.02801 | 0.1774 | 41.49 | 148.32 |
| 100 | Regulation of Microglial Cell Migration (GO:1904139) | 0.02801 | 0.1774 | 41.49 | 148.32 |
| 101 | Regulation of Natural Killer Cell Chemotaxis (GO:2000501) | 0.02801 | 0.1774 | 41.49 | 148.32 |
| 102 | Polysaccharide Biosynthetic Process (GO:0000271) | 0.02801 | 0.1774 | 41.49 | 148.32 |
| 103 | Intracellular Iron Ion Homeostasis (GO:0006879) | 0.03106 | 0.1810 | 7.62 | 26.44 |
| 104 | Negative Regulation of T Cell Mediated Cytotoxicity (GO:0001915) | 0.03195 | 0.1810 | 35.56 | 122.44 |
| 105 | Positive Regulation of Astrocyte Differentiation (GO:0048711) | 0.03195 | 0.1810 | 35.56 | 122.44 |
| 106 | Regulation of Aggrephagy (GO:1905335) | 0.03195 | 0.1810 | 35.56 | 122.44 |
| 107 | Heart Induction (GO:0003129) | 0.03195 | 0.1810 | 35.56 | 122.44 |
| 108 | Positive Regulation of Glial Cell Migration (GO:1903977) | 0.03195 | 0.1810 | 35.56 | 122.44 |
| 109 | Positive Regulation of Lipopolysaccharide-Mediated Signaling Pathway (GO:0031666) | 0.03195 | 0.1810 | 35.56 | 122.44 |
| 110 | Interleukin-5-mediated Signaling Pathway (GO:0038043) | 0.03195 | 0.1810 | 35.56 | 122.44 |
| 111 | Negative Regulation of Protein-Containing Complex Disassembly (GO:0043242) | 0.03195 | 0.1810 | 35.56 | 122.44 |
| 112 | Regulation of Extracellular Matrix Constituent Secretion (GO:0003330) | 0.03195 | 0.1810 | 35.56 | 122.44 |
| 113 | Lung Vasculature Development (GO:0060426) | 0.03195 | 0.1810 | 35.56 | 122.44 |
| 114 | Pericardium Development (GO:0060039) | 0.03195 | 0.1810 | 35.56 | 122.44 |
| 115 | Proton Motive Force-Driven ATP Synthesis (GO:0015986) | 0.03276 | 0.1840 | 7.39 | 25.26 |
| 116 | Oxidative Phosphorylation (GO:0006119) | 0.03449 | 0.1884 | 7.18 | 24.17 |
| 117 | Positive Regulation of Response to External Stimulus (GO:0032103) | 0.03468 | 0.1884 | 4.39 | 14.76 |
| 118 | Release of Sequestered Calcium Ion into Cytosol by Sarcoplasmic Reticulum (GO:0014808) | 0.03587 | 0.1884 | 31.11 | 103.53 |
| 119 | Positive Regulation of Intracellular Estrogen Receptor Signaling Pathway (GO:0033148) | 0.03587 | 0.1884 | 31.11 | 103.53 |
| 120 | Positive Regulation of Macrophage Cytokine Production (GO:0060907) | 0.03587 | 0.1884 | 31.11 | 103.53 |
| 121 | Spermidine Metabolic Process (GO:0008216) | 0.03587 | 0.1884 | 31.11 | 103.53 |
| 122 | Regulation of Heart Morphogenesis (GO:2000826) | 0.03587 | 0.1884 | 31.11 | 103.53 |
| 123 | Determination of Left/Right Asymmetry in Lateral Mesoderm (GO:0003140) | 0.03587 | 0.1884 | 31.11 | 103.53 |
| 124 | Aerobic Electron Transport Chain (GO:0019646) | 0.03807 | 0.1967 | 6.79 | 22.19 |
| 125 | Mitochondrial ATP Synthesis Coupled Electron Transport (GO:0042775) | 0.03807 | 0.1967 | 6.79 | 22.19 |
| 126 | Granulocyte Migration (GO:0097530) | 0.03978 | 0.1977 | 27.65 | 89.16 |
| 127 | Regulation of Cardiac Muscle Cell Differentiation (GO:2000725) | 0.03978 | 0.1977 | 27.65 | 89.16 |
| 128 | Lateral Mesoderm Development (GO:0048368) | 0.03978 | 0.1977 | 27.65 | 89.16 |
| 129 | Regulation of Odontoblast Differentiation (GO:1901329) | 0.03978 | 0.1977 | 27.65 | 89.16 |
| 130 | Regulation of Phosphatase Activity (GO:0010921) | 0.03978 | 0.1977 | 27.65 | 89.16 |
| 131 | Cellular Response to Type I Interferon (GO:0071357) | 0.04178 | 0.2015 | 6.44 | 20.45 |
| 132 | Positive Regulation of Cellular Senescence (GO:2000774) | 0.04367 | 0.2015 | 24.89 | 77.92 |
| 133 | Release of Sequestered Calcium Ion into Cytosol by Endoplasmic Reticulum (GO:1903514) | 0.04367 | 0.2015 | 24.89 | 77.92 |
| 134 | Response to Sterol (GO:0036314) | 0.04367 | 0.2015 | 24.89 | 77.92 |
| 135 | Positive Regulation of Intracellular Steroid Hormone Receptor Signaling Pathway (GO:0033145) | 0.04367 | 0.2015 | 24.89 | 77.92 |
| 136 | Cellular Response to Increased Oxygen Levels (GO:0036295) | 0.04367 | 0.2015 | 24.89 | 77.92 |
| 137 | Negative Regulation of Smooth Muscle Cell Apoptotic Process (GO:0034392) | 0.04367 | 0.2015 | 24.89 | 77.92 |
| 138 | Cellular Response to Oxygen Levels (GO:0071453) | 0.04367 | 0.2015 | 24.89 | 77.92 |
| 139 | Protein Deacetylation (GO:0006476) | 0.04367 | 0.2015 | 24.89 | 77.92 |
| 140 | NK T Cell Activation (GO:0051132) | 0.04367 | 0.2015 | 24.89 | 77.92 |
| 141 | Neuropeptide Signaling Pathway (GO:0007218) | 0.04464 | 0.2016 | 6.20 | 19.28 |
| 142 | Positive Regulation of TOR Signaling (GO:0032008) | 0.04561 | 0.2016 | 6.12 | 18.91 |
| 143 | Negative Regulation of Alcohol Biosynthetic Process (GO:1902931) | 0.04754 | 0.2016 | 22.62 | 68.91 |
| 144 | Golgi Lumen Acidification (GO:0061795) | 0.04754 | 0.2016 | 22.62 | 68.91 |
| 145 | Positive Regulation of Gene Expression via Chromosomal CpG Island Demethylation (GO:0044029) | 0.04754 | 0.2016 | 22.62 | 68.91 |
| 146 | Negative Regulation of Steroid Biosynthetic Process (GO:0010894) | 0.04754 | 0.2016 | 22.62 | 68.91 |
| 147 | Regulation of Leukocyte Proliferation (GO:0070663) | 0.04754 | 0.2016 | 22.62 | 68.91 |
| 148 | Trans-synaptic Signaling (GO:0099537) | 0.04754 | 0.2016 | 22.62 | 68.91 |
| 149 | Modulation by Symbiont of Entry into Host (GO:0052372) | 0.04754 | 0.2016 | 22.62 | 68.91 |
| 150 | Endosomal Lumen Acidification (GO:0048388) | 0.04754 | 0.2016 | 22.62 | 68.91 |
| 151 | Pos Reg Transmembrane Rec Protein Serine/Threonine Kinase Signaling Pathway (GO:0090100) | 0.04758 | 0.2016 | 5.98 | 18.21 |
| 152 | Regulation of Astrocyte Differentiation (GO:0048710) | 0.05140 | 0.2016 | 20.74 | 61.55 |
| 153 | Base-excision Repair, Gap-Filling (GO:0006287) | 0.05140 | 0.2016 | 20.74 | 61.55 |
| 154 | Response to Misfolded Protein (GO:0051788) | 0.05140 | 0.2016 | 20.74 | 61.55 |
| 155 | Host-mediated Suppression of Viral Transcription (GO:0043922) | 0.05140 | 0.2016 | 20.74 | 61.55 |
| 156 | Sarcoplasmic Reticulum Calcium Ion Transport (GO:0070296) | 0.05140 | 0.2016 | 20.74 | 61.55 |
| 157 | Regulation of Dendritic Cell Differentiation (GO:2001198) | 0.05140 | 0.2016 | 20.74 | 61.55 |
| 158 | T Cell Chemotaxis (GO:0010818) | 0.05140 | 0.2016 | 20.74 | 61.55 |
| 159 | Macrophage Chemotaxis (GO:0048246) | 0.05140 | 0.2016 | 20.74 | 61.55 |
| 160 | Nodal Signaling Pathway (GO:0038092) | 0.05140 | 0.2016 | 20.74 | 61.55 |
| 161 | V(D)J Recombination (GO:0033151) | 0.05140 | 0.2016 | 20.74 | 61.55 |
| 162 | Regulation of Natural Killer Cell Mediated Immunity (GO:0002715) | 0.05140 | 0.2016 | 20.74 | 61.55 |
| 163 | Regulation of Sensory Perception (GO:0051931) | 0.05140 | 0.2016 | 20.74 | 61.55 |
| 164 | Positive Regulation of Cell Adhesion (GO:0045785) | 0.05160 | 0.2016 | 5.71 | 16.91 |
| 165 | Negative Regulation of Bone Mineralization (GO:0030502) | 0.05525 | 0.2016 | 19.14 | 55.43 |
| 166 | Positive Regulation of Calcium Ion Import (GO:0090280) | 0.05525 | 0.2016 | 19.14 | 55.43 |
| 167 | Forebrain Neuron Differentiation (GO:0021879) | 0.05525 | 0.2016 | 19.14 | 55.43 |
| 168 | Atrioventricular Valve Development (GO:0003171) | 0.05525 | 0.2016 | 19.14 | 55.43 |
| 169 | Cardiocyte Differentiation (GO:0035051) | 0.05525 | 0.2016 | 19.14 | 55.43 |
| 170 | Regulation of Exit from Mitosis (GO:0007096) | 0.05525 | 0.2016 | 19.14 | 55.43 |
| 171 | Trans-synaptic Signaling, Modulating Synaptic Transmission (GO:0099550) | 0.05525 | 0.2016 | 19.14 | 55.43 |
| 172 | Macrophage Migration (GO:1905517) | 0.05525 | 0.2016 | 19.14 | 55.43 |
| 173 | Magnesium Ion Transport (GO:0015693) | 0.05525 | 0.2016 | 19.14 | 55.43 |
| 174 | Regulation of Macrophage Cytokine Production (GO:0010935) | 0.05525 | 0.2016 | 19.14 | 55.43 |
| 175 | Maintenance of Synapse Structure (GO:0099558) | 0.05525 | 0.2016 | 19.14 | 55.43 |
| 176 | Regulation of Peroxisome Proliferator Activated Receptor Signaling Pathway (GO:0035358) | 0.05525 | 0.2016 | 19.14 | 55.43 |
| 177 | Polyamine Metabolic Process (GO:0006595) | 0.05525 | 0.2016 | 19.14 | 55.43 |
| 178 | Cellular Respiration (GO:0045333) | 0.05680 | 0.2038 | 5.40 | 15.48 |
| 179 | Positive Regulation of Plasma Membrane Bounded Cell Projection Assembly (GO:0120034) | 0.05680 | 0.2038 | 5.40 | 15.48 |
| 180 | Regulation of Innate Immune Response (GO:0045088) | 0.05680 | 0.2038 | 5.40 | 15.48 |
| 181 | Regulation of Trophoblast Cell Migration (GO:1901163) | 0.05908 | 0.2060 | 17.77 | 50.28 |
| 182 | Axonal Transport of Mitochondrion (GO:0019896) | 0.05908 | 0.2060 | 17.77 | 50.28 |
| 183 | T Cell Differentiation in Thymus (GO:0033077) | 0.05908 | 0.2060 | 17.77 | 50.28 |
| 184 | Telomere Maintenance in Response to DNA Damage (GO:0043247) | 0.05908 | 0.2060 | 17.77 | 50.28 |
