# Supplementary material for: Pan-cancer profiles of mesothelin (MSLN) unveil diagnostic potential and therapeutic targetability of pancreatic adenocarcinoma
Source: PLoS One. 2026 Aug 20;21(8):e0354334. doi: 10.1371/journal.pone.0354334 (PMC13492821; doi:10.1371/journal.pone.0354334)
Supplement: Supplementary file 6 [file pone.0354334.s007.docx]

**Table S7.** Molecular functions are associated with MSLN-interacted proteins.

| **Index** | **Name** | **P-value** | **Adjusted p-value** | **Odds Ratio** | **Combined score** |
| --- | --- | --- | --- | --- | --- |
| 1 | Exopeptidase Activity (GO:0008238) | 0.001368 | 0.06414 | 14.98 | 98.81 |
| 2 | DNA Endonuclease Activity, Producing 5'-Phosphomonoesters (GO:0016888) | 0.001428 | 0.06414 | 42.00 | 275.14 |
| 3 | Cytokine Activity (GO:0005125) | 0.001469 | 0.06414 | 6.49 | 42.31 |
| 4 | Receptor Ligand Activity (GO:0048018) | 0.002431 | 0.07962 | 4.12 | 24.80 |
| 5 | DNA Nuclease Activity (GO:0004536) | 0.003879 | 0.1016 | 23.99 | 133.19 |
| 6 | Aminopeptidase Activity (GO:0004177) | 0.007427 | 0.1461 | 16.78 | 82.29 |
| 7 | Hexosyltransferase Activity (GO:0016758) | 0.007806 | 0.1461 | 7.86 | 38.14 |
| 8 | Deoxyribonuclease I Activity (GO:0004530) | 0.02009 | 0.2349 | 62.23 | 243.19 |
| 9 | RNA-DNA Hybrid Ribonuclease Activity (GO:0004523) | 0.02009 | 0.2349 | 62.23 | 243.19 |
| 10 | Double-stranded DNA Exodeoxyribonuclease Activity (GO:0008309) | 0.02009 | 0.2349 | 62.23 | 243.19 |
| 11 | CCR5 Chemokine Receptor Binding (GO:0031730) | 0.02009 | 0.2349 | 62.23 | 243.19 |
| 12 | 4-galactosyl-N-acetylglucosaminide 3-alpha-L-fucosyltransferase Activity (GO:0017083) | 0.02406 | 0.2349 | 49.78 | 185.56 |
| 13 | CCR1 Chemokine Receptor Binding (GO:0031726) | 0.02406 | 0.2349 | 49.78 | 185.56 |
| 14 | Guanyl Nucleotide Exchange Factor Activator Activity (GO:0160124) | 0.02801 | 0.2349 | 41.49 | 148.32 |
| 15 | Alpha-(1->3)-fucosyltransferase Activity (GO:0046920) | 0.03195 | 0.2349 | 35.56 | 122.44 |
| 16 | Omega Peptidase Activity (GO:0008242) | 0.03195 | 0.2349 | 35.56 | 122.44 |
| 17 | UDP-glycosyltransferase Activity (GO:0008194) | 0.03276 | 0.2349 | 7.39 | 25.26 |
| 18 | Morphogen Activity (GO:0016015) | 0.03587 | 0.2349 | 31.11 | 103.53 |
| 19 | Xylosyltransferase Activity (GO:0042285) | 0.03587 | 0.2349 | 31.11 | 103.53 |
| 20 | Double-stranded Telomeric DNA Binding (GO:0003691) | 0.03587 | 0.2349 | 31.11 | 103.53 |
| 21 | UDP-xylosyltransferase Activity (GO:0035252) | 0.03978 | 0.2349 | 27.65 | 89.16 |
| 22 | Ubiquitin Protein Ligase Activity (GO:0061630) | 0.04298 | 0.2349 | 3.17 | 9.98 |
| 23 | Activin Receptor Binding (GO:0070697) | 0.04367 | 0.2349 | 24.89 | 77.92 |
| 24 | Fucosyltransferase Activity (GO:0008417) | 0.04367 | 0.2349 | 24.89 | 77.92 |
| 25 | Natural Killer Cell Lectin-Like Receptor Binding (GO:0046703) | 0.04754 | 0.2349 | 22.62 | 68.91 |
| 26 | BMP Receptor Binding (GO:0070700) | 0.04754 | 0.2349 | 22.62 | 68.91 |
| 27 | Ubiquitin-like Protein Ligase Activity (GO:0061659) | 0.04841 | 0.2349 | 3.05 | 9.22 |
| 28 | DNA-(apurinic or Apyrimidinic Site) Endonuclease Activity (GO:0003906) | 0.05140 | 0.2405 | 20.74 | 61.55 |
| 29 | Carbonate Dehydratase Activity (GO:0004089) | 0.05525 | 0.2496 | 19.14 | 55.43 |
| 30 | Lipase Activator Activity (GO:0060229) | 0.05908 | 0.2496 | 17.77 | 50.28 |
| 31 | Magnesium Ion Transmembrane Transporter Activity (GO:0015095) | 0.05908 | 0.2496 | 17.77 | 50.28 |
