# Supplementary material for: Pan-cancer profiles of mesothelin (MSLN) unveil diagnostic potential and therapeutic targetability of pancreatic adenocarcinoma
Source: PLoS One. 2026 Aug 20;21(8):e0354334. doi: 10.1371/journal.pone.0354334 (PMC13492821; doi:10.1371/journal.pone.0354334)
Supplement: Supplementary file 7 [file pone.0354334.s008.docx]

**Table S8.** Cellular components are associated with MSLN-interacted proteins.

| **Index** | **Name** | **P-value** | **Adjusted p-value** | **Odds Ratio** | **Combined score** |
| --- | --- | --- | --- | --- | --- |
| 1 | PRC1 Complex (GO:0035102) | 0.002376 | 0.2301 | 31.49 | 190.28 |
| 2 | Mitochondrial Inner Membrane (GO:0005743) | 0.008421 | 0.2301 | 3.63 | 17.36 |
| 3 | Multivesicular Body (GO:0005771) | 0.01817 | 0.2301 | 10.27 | 41.15 |
| 4 | Bleb (GO:0032059) | 0.02009 | 0.2301 | 62.23 | 243.19 |
| 5 | Specific Granule Lumen (GO:0035580) | 0.02619 | 0.2301 | 8.38 | 30.52 |
| 6 | Specific Granule (GO:0042581) | 0.02723 | 0.2301 | 4.84 | 17.45 |
| 7 | Alveolar Lamellar Body (GO:0097208) | 0.02801 | 0.2301 | 41.49 | 148.32 |
| 8 | Multivesicular Body Lumen (GO:0097486) | 0.02801 | 0.2301 | 41.49 | 148.32 |
| 9 | Lysosomal Proton-Transporting V-type ATPase Complex (GO:0046611) | 0.02801 | 0.2301 | 41.49 | 148.32 |
| 10 | Cis-Golgi Network (GO:0005801) | 0.02858 | 0.2301 | 7.98 | 28.37 |
| 11 | Late Endosome Lumen (GO:0031906) | 0.03587 | 0.2301 | 31.11 | 103.53 |
| 12 | Cytoplasmic Vesicle Membrane (GO:0030659) | 0.03767 | 0.2301 | 2.84 | 9.31 |
| 13 | FNIP-folliculin RagC/D GAP (GO:1990877) | 0.03978 | 0.2301 | 27.65 | 89.16 |
| 14 | Mitochondrial Membrane (GO:0031966) | 0.04165 | 0.2301 | 2.49 | 7.90 |
| 15 | Clathrin-coated Vesicle (GO:0030136) | 0.04561 | 0.2301 | 6.12 | 18.91 |
| 16 | Vesicle Membrane (GO:0012506) | 0.04561 | 0.2301 | 6.12 | 18.91 |
| 17 | Clathrin-coated Endocytic Vesicle (GO:0045334) | 0.04659 | 0.2301 | 6.05 | 18.55 |
| 18 | Aggresome (GO:0016235) | 0.04754 | 0.2301 | 22.62 | 68.91 |
| 19 | Proton-transporting V-type ATPase, V1 Domain (GO:0033180) | 0.04754 | 0.2301 | 22.62 | 68.91 |
| 20 | Vacuolar Proton-Transporting V-type ATPase, V1 Domain (GO:0000221) | 0.04754 | 0.2301 | 22.62 | 68.91 |
| 21 | Cytoplasmic Stress Granule (GO:0010494) | 0.04758 | 0.2301 | 5.98 | 18.21 |
| 22 | Cis-Golgi Network Membrane (GO:0033106) | 0.05140 | 0.2301 | 20.74 | 61.55 |
| 23 | Lamellar Body (GO:0042599) | 0.05140 | 0.2301 | 20.74 | 61.55 |
| 24 | Plasma Membrane Raft (GO:0044853) | 0.05160 | 0.2301 | 5.71 | 16.91 |
