# Supplementary material for: Pan-cancer profiles of mesothelin (MSLN) unveil diagnostic potential and therapeutic targetability of pancreatic adenocarcinoma
Source: PLoS One. 2026 Aug 20;21(8):e0354334. doi: 10.1371/journal.pone.0354334 (PMC13492821; doi:10.1371/journal.pone.0354334)
Supplement: Supplementary file 8 [file pone.0354334.s009.docx]

**Table S9.** Reactome pathways are associated with the MSLN-interacted genes.

| **Index** | **Name** | **P-value** | **Adjusted p-value** | **Odds Ratio** | **Combined score** |
| --- | --- | --- | --- | --- | --- |
| 1 | SUMOylation of DNA Methylation Proteins | 0.001874 | 0.2154 | 35.99 | 226.04 |
| 2 | Immune System | 0.005052 | 0.2154 | 2.21 | 11.71 |
| 3 | Mitochondrial Translation Elongation | 0.006019 | 0.2154 | 8.67 | 44.32 |
| 4 | Mitochondrial Translation Initiation | 0.006019 | 0.2154 | 8.67 | 44.32 |
| 5 | Mitochondrial Translation Termination | 0.006019 | 0.2154 | 8.67 | 44.32 |
| 6 | TRAF6 Mediated IRF7 Activation | 0.006127 | 0.2154 | 18.65 | 95.03 |
| 7 | Post-translational Modification Synthesis of GPI-anchored Proteins | 0.006392 | 0.2154 | 8.47 | 42.82 |
| 8 | Mitochondrial Translation | 0.007179 | 0.2154 | 8.11 | 40.04 |
| 9 | Transcriptional Regulation by E2F6 | 0.008356 | 0.2228 | 15.73 | 75.28 |
| 10 | Evasion by RSV of Host Interferon Responses | 0.009843 | 0.2362 | 14.38 | 66.46 |
| 11 | SUMOylation of Transcription Cofactors | 0.01373 | 0.2995 | 11.98 | 51.38 |
| 12 | SUMOylation of RNA Binding Proteins | 0.01557 | 0.3113 | 11.18 | 46.54 |
| 13 | Innate Immune System | 0.01739 | 0.3179 | 2.32 | 9.41 |
| 14 | SARS-CoV-2 Activates Modulates Innate and Adaptive Immune Responses | 0.01855 | 0.3179 | 5.64 | 22.48 |
| 15 | Amino Acids Regulate mTORC1 | 0.02094 | 0.3302 | 9.49 | 36.69 |
| 16 | SUMOylation of Chromatin Organization Proteins | 0.02313 | 0.3302 | 8.98 | 33.82 |
| 17 | Regulation of PTEN Gene Transcription | 0.02541 | 0.3302 | 8.52 | 31.30 |
| 18 | Cellular Response to Starvation | 0.02766 | 0.3302 | 4.81 | 17.26 |
| 19 | Complex I Biogenesis | 0.03106 | 0.3302 | 7.62 | 26.44 |
| 20 | POLB-Dependent Long Patch Base Excision Repair | 0.03195 | 0.3302 | 35.56 | 122.44 |
| 21 | Defective CSF2RA Causes SMDP4 | 0.03195 | 0.3302 | 35.56 | 122.44 |
| 22 | Defective CSF2RB Causes SMDP5 | 0.03195 | 0.3302 | 35.56 | 122.44 |
| 23 | Translation | 0.03428 | 0.3302 | 3.42 | 11.54 |
| 24 | Displacement of DNA Glycosylase by APEX1 | 0.03587 | 0.3302 | 31.11 | 103.53 |
| 25 | Cytokine Signaling in Immune System | 0.03767 | 0.3302 | 2.36 | 7.72 |
| 26 | SUMOylation of DNA Damage Response and Repair Proteins | 0.03898 | 0.3302 | 6.70 | 21.73 |
| 27 | Reactions Specific to the Complex N-glycan Synthesis Pathway | 0.03978 | 0.3302 | 27.65 | 89.16 |
| 28 | Diseases Associated with Surfactant Metabolism | 0.03978 | 0.3302 | 27.65 | 89.16 |
| 29 | Interferon Alpha Beta Signaling | 0.03990 | 0.3302 | 6.61 | 21.29 |
| 30 | Endosomal Vacuolar Pathway | 0.04754 | 0.3359 | 22.62 | 68.91 |
| 31 | Reversible Hydration of Carbon Dioxide | 0.04754 | 0.3359 | 22.62 | 68.91 |
| 32 | Signaling by LTK | 0.04754 | 0.3359 | 22.62 | 68.91 |
| 33 | Regulation of Signaling by NODAL | 0.04754 | 0.3359 | 22.62 | 68.91 |
| 34 | DDX58 IFIH1-mediated Induction of Interferon-Alpha Beta | 0.04758 | 0.3359 | 5.98 | 18.21 |
| 35 | p38MAPK Events | 0.05140 | 0.3427 | 20.74 | 61.55 |
| 36 | NF-kB Activation Through FADD RIP-1 Pathway Mediated by Caspase-8 and -10 | 0.05140 | 0.3427 | 20.74 | 61.55 |
| 37 | Antimicrobial Peptides | 0.05680 | 0.3594 | 5.40 | 15.48 |
| 38 | SARS-CoV-2-host Interactions | 0.05871 | 0.3594 | 3.52 | 9.99 |
