# Supplementary material for: Pan-cancer profiles of mesothelin (MSLN) unveil diagnostic potential and therapeutic targetability of pancreatic adenocarcinoma
Source: PLoS One. 2026 Aug 20;21(8):e0354334. doi: 10.1371/journal.pone.0354334 (PMC13492821; doi:10.1371/journal.pone.0354334)
Supplement: Supplementary file 9 [file pone.0354334.s010.docx]

**Table S10.** Wiki pathways are associated with MSLN-interacted genes.

| **Index** | **Name** | **P-value** | **Adjusted p-value** | **Odds Ratio** | **Combined score** |
| --- | --- | --- | --- | --- | --- |
| 1 | Interactions Of Natural Killer Cells in Pancreatic Cancer WP5092 | 0.005719 | 0.2617 | 19.37 | 100.02 |
| 2 | Interferon Mediated Signaling WP4558 | 0.009843 | 0.2617 | 14.38 | 66.46 |
| 3 | Overview Of Proinflammatory and Profibrotic Mediators WP5095 | 0.01552 | 0.2617 | 6.04 | 25.17 |
| 4 | Cytokine Cytokine Receptor Interaction WP5473 | 0.01999 | 0.2617 | 4.07 | 15.93 |
| 5 | Mitochondrial Complex I Assembly Model OXPHOS System WP4324 | 0.02166 | 0.2617 | 9.31 | 35.69 |
| 6 | Lacto Series Sphingolipid Metabolism WP5303 | 0.02406 | 0.2617 | 49.78 | 185.56 |
| 7 | Novel Intracellular Components of RIG I Like Receptor Pathway WP3865 | 0.02464 | 0.2617 | 8.67 | 32.11 |
| 8 | Oxidative Phosphorylation WP623 | 0.02464 | 0.2617 | 8.67 | 32.11 |
| 9 | Lung Fibrosis WP3624 | 0.02698 | 0.2617 | 8.24 | 29.77 |
| 10 | SARS CoV 2 Innate Immunity Evasion and Cell Immune Response WP5039 | 0.02940 | 0.2617 | 7.85 | 27.70 |
| 11 | Tgif Disruption of Shh Signaling WP3674 | 0.03587 | 0.2690 | 31.11 | 103.53 |
| 12 | Cytosolic DNA Sensing Pathway WP4655 | 0.03626 | 0.2690 | 6.98 | 23.15 |
| 13 | PtdIns 4 5 P2 In Cytokinesis Pathway WP5199 | 0.04754 | 0.2821 | 22.62 | 68.91 |
| 14 | Bone Morphogenic Protein Signaling and Regulation WP1425 | 0.04754 | 0.2821 | 22.62 | 68.91 |
| 15 | RalA Downstream Regulated Genes WP2290 | 0.04754 | 0.2821 | 22.62 | 68.91 |
| 16 | BMP2 WNT4 FOXO1 Pathway Primary Endometrial Stromal Cell Diff WP3876 | 0.05140 | 0.2859 | 20.74 | 61.55 |
| 17 | Toll Like Receptor Signaling WP75 | 0.05680 | 0.2912 | 5.40 | 15.48 |
| 18 | COVID 19 Adverse Outcome Pathway WP4891 | 0.05908 | 0.2912 | 17.77 | 50.28 |
