# Supplementary material for: Pan-cancer profiles of mesothelin (MSLN) unveil diagnostic potential and therapeutic targetability of pancreatic adenocarcinoma
Source: PLoS One. 2026 Aug 20;21(8):e0354334. doi: 10.1371/journal.pone.0354334 (PMC13492821; doi:10.1371/journal.pone.0354334)
Supplement: Supplementary file 10 [file pone.0354334.s011.docx]

**Table S11.** KEGG pathways are associated with the MSLN-interacted proteins.

| **Index** | **Name** | **P-value** | **Adjusted p-value** | **Odds Ratio** | **Combined score** |
| --- | --- | --- | --- | --- | --- |
| 1 | CYTOKINE-CYTOKINE RECEPTOR INTERACTION | 0.001280 | 0.1020 | 5.41 | 36.07 |
| 2 | NATURAL KILLER CELL MEDIATED CYTOTOXICITY | 0.001981 | 0.1020 | 8.10 | 50.39 |
| 3 | RIG-I-LIKE RECEPTOR SIGNALING PATHWAY | 0.003000 | 0.1030 | 11.23 | 65.22 |
| 4 | OTHER TYPES OF O-GLYCAN BIOSYNTHESIS | 0.01314 | 0.3235 | 12.27 | 53.17 |
| 5 | OXIDATIVE PHOSPHORYLATION | 0.01716 | 0.3235 | 5.81 | 23.62 |
| 6 | AUTOIMMUNE THYROID DISEASE | 0.01885 | 0.3235 | 10.06 | 39.95 |
| 7 | RIBOSOME | 0.02723 | 0.3910 | 4.84 | 17.45 |
| 8 | JAK-STAT SIGNALING PATHWAY | 0.03037 | 0.3910 | 4.63 | 16.19 |
| 9 | POLYCOMB REPRESSIVE COMPLEX | 0.04368 | 0.4553 | 6.28 | 19.66 |
| 10 | HUMAN PAPILLOMAVIRUS INFECTION | 0.04420 | 0.4553 | 3.14 | 9.80 |
| 11 | RHEUMATOID ARTHRITIS | 0.05366 | 0.4638 | 5.58 | 16.32 |
