# Supplementary material for: Pan-cancer profiles of mesothelin (MSLN) unveil diagnostic potential and therapeutic targetability of pancreatic adenocarcinoma
Source: PLoS One. 2026 Aug 20;21(8):e0354334. doi: 10.1371/journal.pone.0354334 (PMC13492821; doi:10.1371/journal.pone.0354334)
Supplement: Supplementary file 11 [file pone.0354334.s012.docx]

**Table S12.** GDSC drug-gene correlation analysis for MSLN.

| Drug | Cor | FDR | Association |
| --- | --- | --- | --- |
| AR-42 | 0.142 | 4.97E-05 | Resistant |
| AT-7519 | 0.132 | 0.000171 | Resistant |
| Afatinib | -0.133 | 0.000171 | Sensitive |
| BMS345541 | 0.129 | 0.000251 | Resistant |
| BX-795 | 0.117 | 0.004058 | Resistant |
| Belinostat | 0.132 | 0.000248 | Resistant |
| CAL-101 | 0.088 | 0.018210 | Resistant |
| CAY10603 | 0.167 | 1.46E-06 | Resistant |
| CP466722 | 0.114 | 0.001258 | Resistant |
| CX-5461 | 0.110 | 0.002253 | Resistant |
| Camptothecin | 0.119 | 0.002832 | Resistant |
| Cetuximab | -0.188 | 4.11E-07 | Sensitive |
| Dabrafenib | 0.092 | 0.022330 | Resistant |
| Dasatinib | -0.181 | 0.002812 | Sensitive |
| Erlotinib | -0.232 | 0.000224 | Sensitive |
| FK866 | 0.140 | 7.62E-05 | Resistant |
| GSK1070916 | 0.151 | 2.16E-05 | Resistant |
| GSK2126458 | 0.126 | 0.000514 | Resistant |
| GSK429286A | 0.116 | 0.002317 | Resistant |
| GSK690693 | 0.127 | 0.000387 | Resistant |
| I-BET-762 | 0.140 | 4.79E-05 | Resistant |
| IPA-3 | 0.113 | 0.002918 | Resistant |
| JQ1 | 0.091 | 0.022893 | Resistant |
| JW-7-24-1 | 0.199 | 6.71E-09 | Resistant |
| Lapatinib | -0.190 | 0.001200 | Sensitive |
| Masitinib | 0.162 | 5.17E-06 | Resistant |
| Methotrexate | 0.138 | 0.000138 | Resistant |
| Navitoclax | 0.153 | 2.46E-05 | Resistant |
| OSI-027 | 0.173 | 7.71E-07 | Resistant |
| PI-103 | 0.217 | 2.93E-10 | Resistant |
| PD-0325901 | -0.131 | 0.000598 | Sensitive |
| RDEA119 | -0.127 | 0.000318 | Sensitive |
| Saracatinib | -0.161 | 0.029973 | Sensitive |
| SN-38 | 0.117 | 0.002823 | Resistant |
| TG101348 | 0.141 | 5.63E-05 | Resistant |
| THZ-2-102-1 | 0.182 | 1.73E-07 | Resistant |
| Trametinib | -0.126 | 0.000478 | Sensitive |
| Tubastatin A | 0.134 | 0.000137 | Resistant |
| UNC0638 | 0.193 | 1.03E-08 | Resistant |
| Vinblastine | 0.112 | 0.005621 | Resistant |
| Vorinostat | 0.159 | 1E-05 | Resistant |
| WZ-1-84 | -0.212 | 0.000927 | Sensitive |
| XAV939 | -0.188 | 2.29E-07 | Sensitive |
