# Supplementary material for: Pan-cancer profiles of mesothelin (MSLN) unveil diagnostic potential and therapeutic targetability of pancreatic adenocarcinoma
Source: PLoS One. 2026 Aug 20;21(8):e0354334. doi: 10.1371/journal.pone.0354334 (PMC13492821; doi:10.1371/journal.pone.0354334)
Supplement: Supplementary file 12 [file pone.0354334.s013.docx]

**Table S13.** CTRP drug–gene correlation analysis for MSLN.

| **Drug** | **Cor** | **FDR** | **Association** |
| --- | --- | --- | --- |
| ABT-199 | 0.177 | 0.000715 | Resistant |
| ABT-737 | 0.185 | 7.93E-06 | Resistant |
| AM-580 | 0.151 | 0.001129 | Resistant |
| AT7867 | 0.222 | 8.15E-09 | Resistant |
| AZD1480 | 0.209 | 0.000969 | Resistant |
| AZD4547 | 0.215 | 2.5E-07 | Resistant |
| BI-2536 | 0.256 | 1.33E-12 | Resistant |
| BIX-01294 | 0.209 | 3.08E-08 | Resistant |
| BMS-345541 | 0.197 | 2.74E-07 | Resistant |
| CCT036477 | 0.264 | 1.13E-12 | Resistant |
| CD-437 | 0.203 | 5.33E-08 | Resistant |
| CHM-1 | 0.264 | 5.42E-13 | Resistant |
| Doxorubicin | 0.236 | 7.99E-11 | Resistant |
| Etoposide | 0.256 | 2.98E-12 | Resistant |
| FQI-2 | 0.239 | 6.57E-11 | Resistant |
| GSK461364 | 0.241 | 7.07E-11 | Resistant |
| JQ-1 | 0.199 | 7.95E-08 | Resistant |
| KX2-391 | 0.259 | 2.42E-12 | Resistant |
| LY-2183240 | 0.270 | 1.36E-13 | Resistant |
| MG-132 | 0.240 | 0.000931 | Resistant |
| MST-312 | 0.249 | 2.88E-11 | Resistant |
| PI-103 | 0.223 | 8.91E-09 | Resistant |
| Rigosertib | 0.249 | 3.23E-11 | Resistant |
| SB-225002 | 0.312 | 2.98E-18 | Resistant |
| SB-743921 | 0.210 | 8.45E-09 | Resistant |
| SN-38 | 0.208 | 5.83E-07 | Resistant |
| Sunitinib | 0.214 | 2.4E-08 | Resistant |
| Topotecan | 0.232 | 1.83E-10 | Resistant |
| Vincristine | 0.278 | 5.46E-15 | Resistant |
| Vorinostat | 0.188 | 5.98E-07 | Resistant |
| YK-4-279 | 0.235 | 1.63E-10 | Resistant |
