# Supplementary material for: Pan-cancer profiles of mesothelin (MSLN) unveil diagnostic potential and therapeutic targetability of pancreatic adenocarcinoma
Source: PLoS One. 2026 Aug 20;21(8):e0354334. doi: 10.1371/journal.pone.0354334 (PMC13492821; doi:10.1371/journal.pone.0354334)
Supplement: Supplementary file 13 [file pone.0354334.s014.docx]

**Table S14.** Binding affinities of all complexes and phytochemicals class.

| **CID** | **Phytochemicals** | **Phytochemical class** | **Binding Affinity (kcal/mol)** |
| --- | --- | --- | --- |
| 265237 | Withaferin A | Steroidal lactone (Withanolide) | -8.0 |
| 10494 | Oleanolic acid | Pentacyclic triterpenoid | -7.7 |
| 64945 | Ursolic acid | Pentacyclic triterpenoid | -7.6 |
| 5280805 | Rutin | Flavonoid glycoside (Flavonol glycoside) | -7.5 |
| 10281 | Thymoquinone | Monoterpene quinone | -5.0 |
| 10621 | Hesperidin | Flavanone glycoside | -7.3 |
| 168928 | Pyrimidine derivatives | Pyrimidine alkaloid / Heterocyclic compound | -7.2 |
| 2353 | Berberine | Isoquinoline alkaloid | -6.4 |
| 3220 | Emodin | Anthraquinone | -6.6 |
| 370 | Gallic acid | Phenolic acid | -5.4 |
| 445154 | Resveratrol | Stilbene polyphenol | -6.6 |
| 5280343 | Quercetin | Flavonol | -7.1 |
| 5280443 | Apigenin | Flavone | -6.9 |
| 5280445 | Luteolin | Flavone | -7.1 |
| 5280863 | Kaempferol | Flavonol | -7.0 |
| 5280961 | Genistein | Isoflavone | -6.9 |
| 5281605 | Baicalein | Flavone | -7.3 |
| 5281654 | Isorhamnetin | O-methylated flavonol | -6.7 |
| 5281672 | Myricetin | Flavonol | -6.9 |
| 5281855 | Ellagic acid | Polyphenolic compound / Phenolic acid | -7.1 |
| 638024 | Piperine | Alkaloid (Piperidine alkaloid) | -7.2 |
| 64971 | Betulinic acid | Pentacyclic triterpenoid | -7.3 |
| 65036 | Allicin | Organosulfur compound | -3.5 |
| 65064 | (-)-Epigallocatechin gallate (EGCG) | Catechin (Flavan-3-ol) | -6.6 |
| 932 | (+)-Naringenin | Flavanone | -6.8 |
| 969516 | Curcumin | Diarylheptanoid / Polyphenol | -6.6 |
| 160254 | Cryptotanshinone | Diterpenoid quinone | -7.4 |
| 222284 | (-)-β-Sitosterol | Phytosterol (Sterol) | -7.1 |
| 5281647 | Mangiferin | Xanthone glycoside | -7.0 |
| 441788 | Bruceine D | Quassinoid (Triterpenoid derivative) | -6.6 |
